# COFACTOR-SBHUB Oslo: Hourly Sub-Metered Energy Use Data from 48 public School Buildings in Oslo, Norway

**DOI:** 10.1016/j.dib.2025.112288

**Published:** 2025-11-15

**Authors:** Synne Krekling Lien, Bjørn Ludvigsen, Harald Taxt Walnum, Aileen Yang, Åse Lekang Sørensen, Kamilla Heimar Johra

**Affiliations:** SINTEF Community**,** NO-0314 Oslo, Norway

**Keywords:** Time series data, Energy use, Heat use, Hourly, Buildings, Schools, Sub-meters

## Abstract

This article describes a dataset of hourly sub-metered energy use data from 48 school buildings located in Oslo, owned and managed by Oslobygg KF. The dataset consists of 1 comma-delimited file per building, each containing meta data about the building, time series data containing energy use measurements and local weather data. The length of the dataset varies by building, covering between 1 and 11 years of raw data. Raw data for each building was downloaded in 2023 from Oslobygg KF’s energy management system, “Energinet.” Only buildings with sufficiently reliable sub-metered heating data were included. This process included manual selection, quality-control, relabelling and cleaning to ensure consistency and accuracy. The dataset includes buildings with both electric heating (electric boilers and/or heat pumps) and district heating. All buildings have sub-metered heating data, and some also include sub-meters for domestic hot water heating and photovoltaic electricity generation. The data set can be used for several research and engineering purposes, including benchmarking and validation of building simulations, heating disaggregation, energy use time series classification, forecasting of energy loads and flexibility, grid planning and other modelling activities.

Specifications Table

**Table:** 

Subject	Engineering & Materials science
Specific subject area	*Hourly energy use measurements from buildings*
Type of data	*Table; Figure; Processed human readable comma seperated .txt files; plots (.png); readme (.txt)*
Data collection	*Hourly energy use data were collected from Oslobygg KF’s Energy Management System Energinet from the company Kiona. Energinet is a software for energy, waste and environmental reporting, where energy meter data is directly imported via SFTP, email, industry protocols (e.g. OBIX), third-party APIs, and directly through Energinet’s API solutions.. Energy use measurements from district heating meters, electricity main meters (AMS) and energy sub-meters from Energinet were downloaded to csv files and relabelled and cleaned. Weather data for each building were sourced from the MET Norway Nordic dataset. Data cover between 1–11 years per building (2012–2024) and were processed using Python (v3.11) and Pandas (v2.2.3) for cleaning and formatting.*
Data source location	*Institution: Schools owned and managed by Oslobygg KF* *City/Town/Region: Oslo municipality* *Country: Norway* *Latitude and longitude: 59° 54′ 45.8" North, 10° 44′ 45.9" East*
Data accessibility	Repository name: data.sintef.no Data identification number (all versions): https://doi.org/10.60609/czgf-5e46 Direct URL to repository: https://data.sintef.no/product/dp-679b0640-834e-46bd-bc8f-8484ca79b414 Instructions for accessing the data: The repository is open access and does not require sign-in.
Related research article	https://doi.org/10.1016/j.enbuild.2025.116359 [1]

## 1 Value of the Data

• The open dataset contains meta data and hourly energy use data from 48 school buildings in Oslo including sub-meters for heating for several years. There are few datasets publicly available with long time series containing building energy data, especially with sub-meters for electric heating appliances. The dataset can improve the knowledge on how school buildings use energy in general and for heating and other purposes. The dataset contains open, structured, and documented time series data compatible with common analysis tools (Python, R, Excel, MatLab).
• Researchers, PhD-candidates and students can use the dataset to train, test, and validate data-driven models for heat load disaggregation in buildings with district heating and electric heating. Disaggregation, or non-intrusive load monitoring (NILM), involves separating the total energy use of a building into end-use components without installing additional sub-meters. Validated disaggregation models can reveal how individual loads and heating technologies contribute to total energy use and peak demand. Such insights can support energy cost reduction for building owners and inform strategies to improve building energy efficiency and reduce strain on the electricity grid. The development of these models depends on access to reliable, high-resolution sub-metered data such as those provided in this dataset. The dataset was used for this purpose in [1] to examine how to disaggregate electricity use for heating in all-electric buildings by training on energy use data from district heating buildings, and is suitable for similar studies.
• For grid planners, district heating operators, DSOs and energy agencies, there is a need to examine how buildings’ energy use contribute to hourly energy use and peaks in the electricity and heating grids, especially with increased electrification of other sectors, such as the industry and transport sector. As the dataset contain data from several different schools in Norway with different sizes, age, heating systems and other configurations, the dataset can be used to generate typical load profiles for school buildings to be used for building load forecasting. As the dataset also contains sub-meter information it is valuable for energy efficiency analysis, scenario analysis and analysis of demand-side flexibility potential. As the buildings are also located in the same location the data can also be used to examine the coincidence factor of school buildings.
• For building owners, facility mangers, building operators and developers of building energy control and management/monitoring systems, the dataset provides detailed sub-metered energy use measurements that can be used for benchmarking of schools, performance analysis, and the development of fault detection and diagnostic models for school buildings.
• For Oslobygg KF, the data provider , and other municipal entities managing public building portfolios, the dataset offers a valuable reference for data quality control, portfolio overview, and evaluation of energy efficiency measures across schools. It can support strategic planning and portfolio-level energy management.

## 2 Background

Electric heating is widespread in Norwegian buildings and significantly contributes to peak loads in the electricity grid [2]. Despite this, most buildings lack sub-meters for electric heating. Disaggregation methods offer alternatives to sub-metering by using data-driven methods to extract electricity use for appliances from time-series data, but require sub-metered data for training and validation [3]. This data descriptor presents a dataset of hourly energy measurements from 48 school buildings in Oslo, which was used for developing a heating disaggregation method in [1]*. Oslo is* Norway’s most populated city, with approximately 700,000 residents [4] and manages 181 public schools with 84 000 students [5,6]. These schools vary significantly in size, architecture, energy efficiency, heating systems, and age, ranging from 19th-century buildings to modern schools built in the 2010s. The school buildings are managed by Oslobygg KF (hereby referred to as “Oslobygg”) which is a municipal enterprise in Oslo, Norway, responsible for planning, building, managing, and maintaining public buildings. The dataset, collected in 2023, results from a collaboration between Oslobygg, the Smart Building Hub (SBHub) project [7], and the COFACTOR project which is a research project aiming to address the knowledge gap in energy use ``behind the meter'' by collecting sub-metered data [8].

## 3 Data Description

The data set consists of 48 school buildings located in Oslo, Norway. The dataset consists of one csv-file per building containing meta data and energy and weather time series data. In addition, a plot is included for each building, providing an overview of the energy time series data. The time series data have a duration of approximately 1-11 years per building with hourly resolution. *This section describes the dataset, and how to find and use this.*

### 3.1 Repository

The data is stored in a repository organized as an open access Data Product in the data.sintef.no data platform (https://data.sintef.no/). A direct link to the repository is here: https://data.sintef.no/product/dp-679b0640-834e-46bd-bc8f-8484ca79b414.

**Fig. 1:**
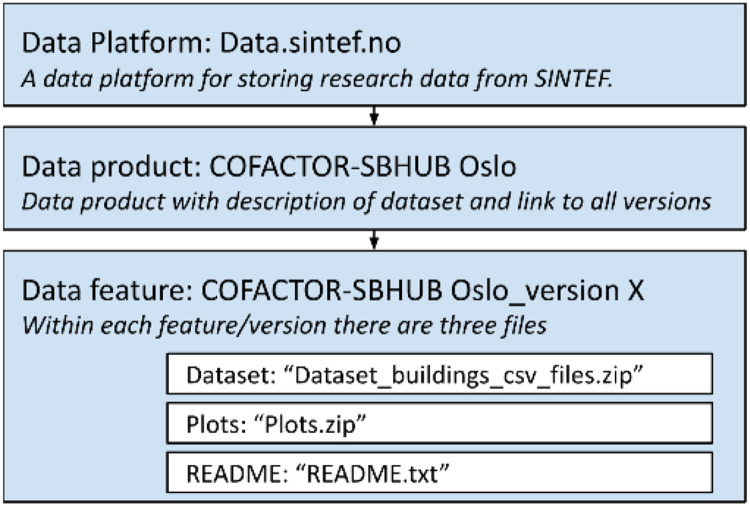
Repository set up.

**Fig. 2:**
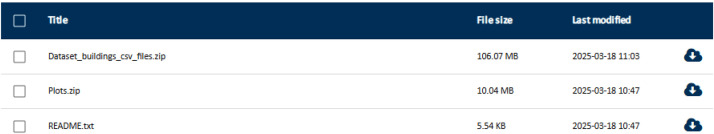
Screen shot of the “data feature” in the repository.

The Data Product contains a description of the dataset and a link to all versions of the Data Feature (the dataset), as described in Fig. 1. A screen show of how the data feature is stored in the reposotiry is shown in Fig. 2. The Data Feature contains three files:

1) Dataset_buildings_csv_files.zip: A zip containing 48 human readable comma separated .txt files, one per building with building meta data and time series data. The time series data contain hourly sub-metered treated energy use data from the 48 school buildings located in Oslo, owned and managed by Oslobygg KF. The csv files are named “export_uniqueID” to preserve anonymity and a unique ID.
2) Plots.zip: ZIP containing 48 .png files with plots of the energy use columns in each building in the csv files, plotted against time.
3) README.txt: Description of dataset, csv-files and abbreviations in the csv files. Fig. 2 shows a Screen shot of how the “data feature” is stored in the repository.

### 3.2 CSV-file structure

**Fig. 3:**
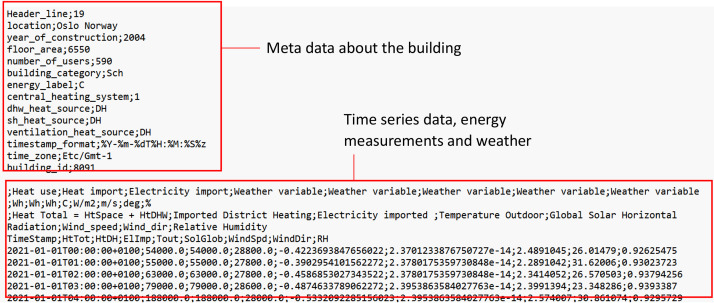
Example of one of the CSV-files per building

An example of one of these CSV-files is shown in Fig. 3. The CSV-files consist of two parts 1) meta data about the building and 2) time series data, and follows the structure of files in the trEASURE database [9]. The meta data contains information about the building, including its size, users, and heating system. The time series data contains 4 rows of headers, including the long name, unit, description and short name. The time series index contains timestamps formatted as %Y-%m-%dT%H:%M:%S%z, following the ISO 8601 standard (https://www.iso.org/standard/70907.html). The timestamps are given in local normal time: CET (UTC/GMT+01:00). The time series is left-labelled, meaning that a timestamp such as 2018-01-01T00:00:00 +01:00 represents the use during the hour 00:00-01:00. The actual local time zone of the location is “Europe/Oslo”.

Table 1 describes all the available meta data parameters, descriptions and abbreviations available in the csv-files.

**Table 1:** Meta data parameters and format.

Meta data parameter	Description	Data type	Example
Header_line	First line of measurements (for reading the csv)	Int	39
location	Post code or location of the building	Str	‘Oslo’
year_of_construction	Year of construction	Int	1958
floor_area	Floor area in square meters	Int	5036
number_of_users	Number of users	Int	200
number_of_buildings	Number of buildings on the lot	Int	2
building_category	Building category abbreviation. School: ‘Sch’	Str	‘Sch’
energy_label	Energy label	Str	‘A’ to ‘G’
notes	Any notes/additional information about the building and energy data	Str	‘Description’
central_heating_system	Buildings with central heating systems and water borne heat distribution systems.	Int [0,1,2]	0 – No 1 – yes 2 – Unknown
dhw_heat_source	Type(s) of heating technology for hot water heating. See table below for available options.	Str	‘HWH’
sh_heat_source	Type(s) of heating technology for space heating. See table below for available options.	Str	‘A2A,EFH,EH’
ventilation_heat_source	Type(s) of heating technology for ventilation heating. See table below for available options.	Str	‘DH’
Pv	Photovoltaic system. Location, size (kWp) and inverter capacity (kW)	Str	‘Roof:3:3, Wall:5:4’
timestamp_format	Format for the timestamp in the time series	Str	‘%Y-%m-%dT%H:%M:%S%z’
time_zone	Time zone	Str	UTC/GMT+01:00
building_id	Id of the building	Str	‘8091’

For heating options in sh_heat_source, dhw_heat_source and ventilation_heat_source, the schools can use one or more of the following heating technologies listed in Table 2.

**Table 2:** Abbreviations of heating types for the different energy services in the file meta data.

Option name	Option description	Available for energy services
EB	Electric boiler	SH, DHW, VH, SM
EFH	Electric floor heater	SH
EH	Electric heater	SH
DH	District heating	SH, DHW, VH, SM
GSHP	Ground source heat pump	SH, DHW, VH, SM
ASHP	Air source heat pump	SH, DHW, VH, SM
SC	Solar collector	SH, DHW, VH, SM
HWH	Hot water heater	DHW
EHB	Electric heating battery	VH
Ukn	Unknown	SH, DHW, VH

SH = Space heating, DHW = domestic hot water heating, VH = ventilation heating, SM = Snow melt systems

To be able to compare measurements of energy purposes from different buildings, the different meters in the energy management system have been assigned to predefined measurement purposes. All possible meter types in the time series data from the building files are described Table 3.

**Table 3:** Time series columns.

Column name	Description	Measurement category	Unit
TimeStamp	Time stamp in local UTC time	Time	Time
Tout	Temperature Outdoor	Weather variable	C
SolGlob	Global Solar Horizontal Radiation	Weather variable	W/m^2^
WindSpd	Wind speed	Weather variable	m/s
WindDir	Wind direction	Weather variable	deg
RH	Relative humidity	Weather variable	%
ElPV	Electricity production	Electricity production from PV	Wh
ElImp	Electricity imported	Electricity Import	Wh
ElLight	Electricity lighting	Electricity use	Wh
ElLightOut	Electricity outdoor lighting	Electricity use	Wh
ElFan	Electricity ventilation fans	Electricity use	Wh
ElOth	Electricity other	Electricity use	Wh
ElDHW	Electricity for domestic hot water heating	Electricity use	Wh
ElBoil	Electricity for electric boiler	Electricity use	Wh
ElHP	Heat pump	Electricity use	Wh
ElTech	Electricity for technical room/server room	Electricity use	Wh
ElSnow	Electricity for snow melt	Electricity use	Wh
ElHWH	Electricity for hot water heater	Electricity use	Wh
ElClComf	Electricity for comfort cooling	Electricity use	Wh
ElClTech	Electricity for technical cooling	Electricity use	Wh
ElPump	Electricity use for pumps	Electricity use	Wh
ElVentPool	Electricity for ventilation of pool area	Electricity use	Wh
ElEV	Electricity for charging of electric vehicles	Electricity use	Wh
HtOil	Heat production from Oil	Heat production	Wh
HtSC	Heat production from Solar collector	Heat production	Wh
HtDH	Heat from district heating	Heat Import	Wh
HtTot	Heat Total HtTot = HtSpace + HtDHW	Heat use	Wh
HtSpace	Space heating HtSpace = HtRoom + HtVent	Heat use	Wh
HtRoom	Room heating	Heat use	Wh
HtVent	Ventilation heating battery	Heat use	Wh
HtDHW	Domestic Hot Water	Heat use	Wh
HtHP	Heat from heat pumps	Heat production	Wh
HtSnow	Snow melting	Heat use	Wh
HtPool	Heat for pool heating	Heat use	Wh

### 3.3 Plots

**Fig. 4:**
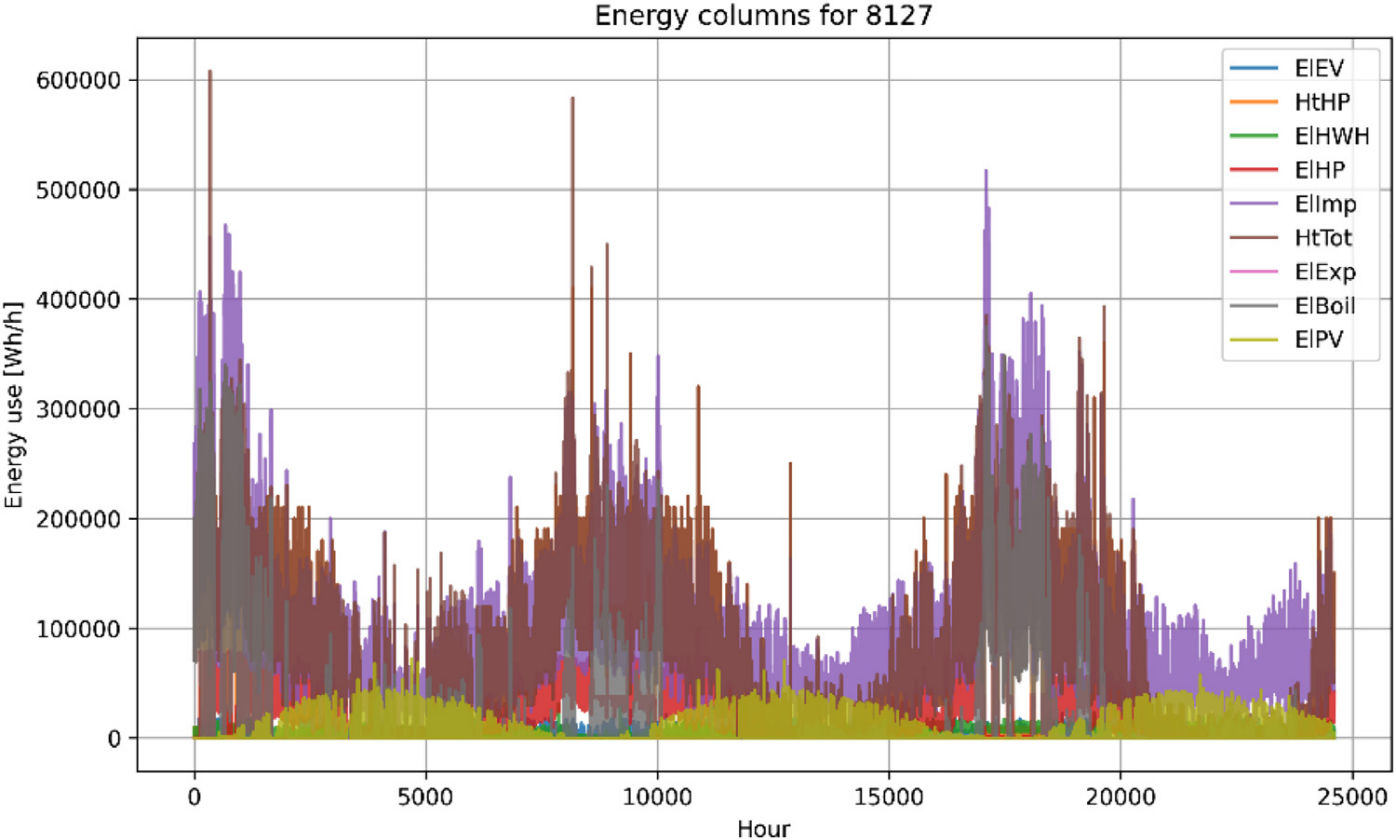
Example plot of energy columns for building 8127.

**Fig. 5:**
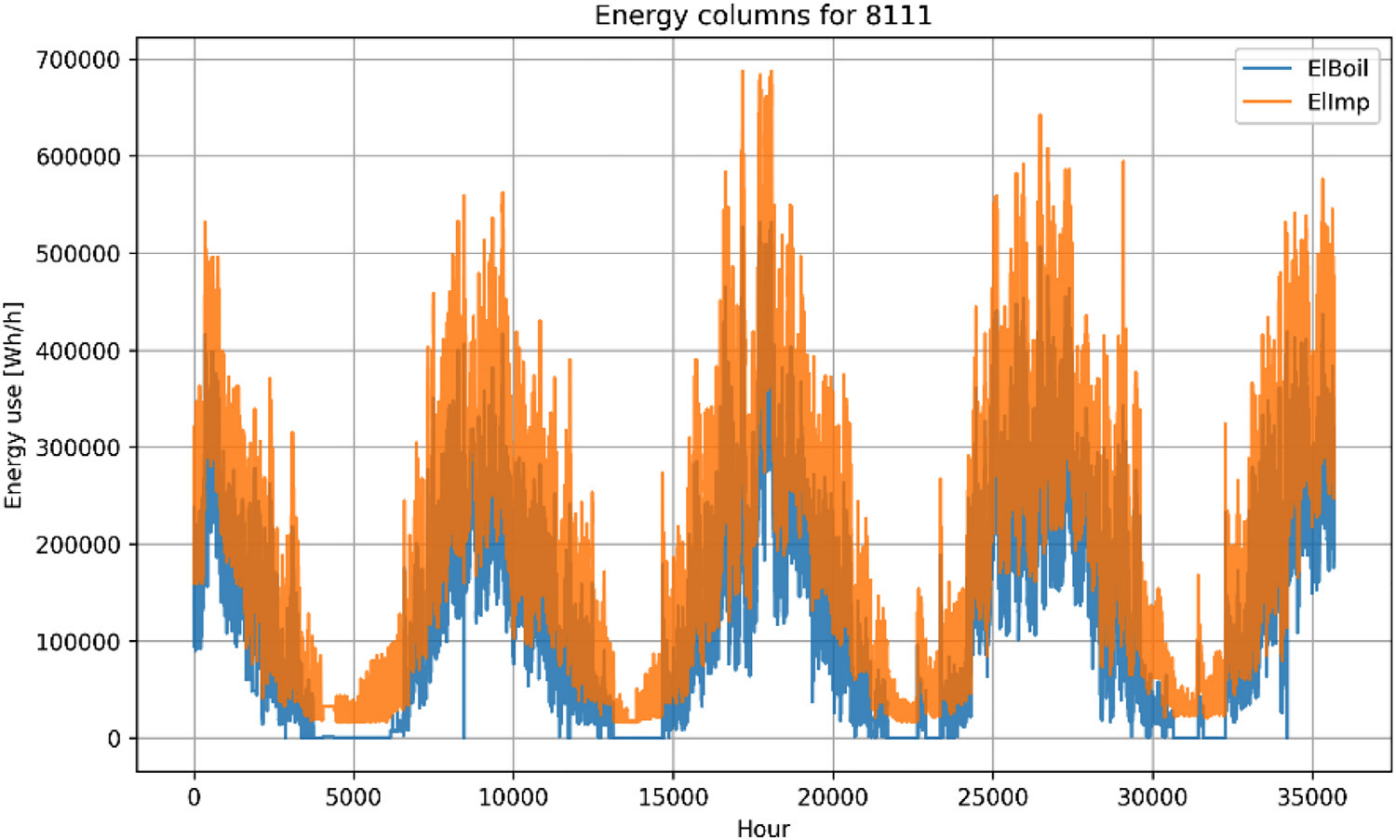
Example plot of energy columns for building 8111.

In addition to the csv-files, the energy columns within each building file have been plotted to give an overview of the energy use, available meters and duration of each building within the “Plots”-folder. Fig. 4, Fig. 5 shows two examples of energy-columns plots from the plots-folder.

### 3.4 Summary of the buildings in the dataset

**Fig. 6:**
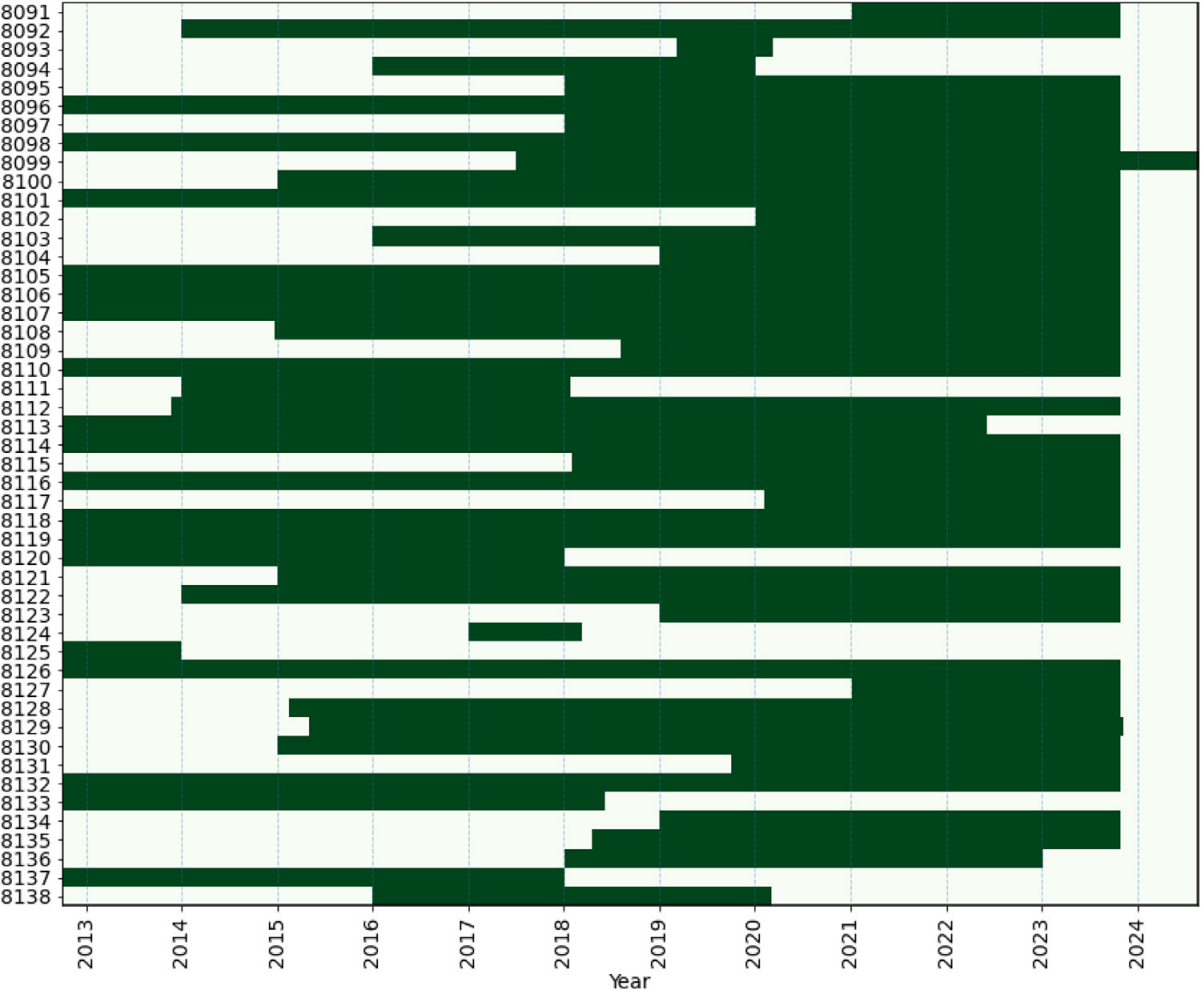
Available data period for each building in the dataset.

A short description of all buildings available in the dataset is given in Table 4, including a summary of the meta data for each building, the duration of the time series data, and the energy columns present in each file. The available time stamps within each file/building is shown in Fig. 6.

**Table 4:** Descriptioon of buildings in the dataset.

ID	Year of const.	Floor area	# users	Dhw heat source	Sh heat source	Years of data	Energy column in file
8091	2004	6550	590	DH	DH	2.8	HtTot, HtDH, ElImp
8092	1970	7047	450	GSHP, BB	GSHP, BB	9.8	ElImp, HtTot, ElHP, HtHP
8093	1965	9072	770	DH	DH	1	HtTot, ElImp
8094		27479	756	DH	DH	4	HtDH, HtTot, ElImp
8095	1975	6066	170	GSHP,HWH, BB	GSHP, BB	5.8	HtBio, ElHP, HtHP, HtTot, ElImp, ElSpace
8096	1898	5820	415	DH	DH	11.1	HtTot, HtDH, ElImp
8097	1972	6481	600	EB, SC, ASHP	EB, SC, ASHP	5.8	ElPH, ElBoil, ElImp, ElHP
8098	1900	6736	400	EB	EB	11.1	ElBoil, ElImp
8099	2017	11680	840	GSHP, HWH	EB, GSHP	7.1	ElSnow, HtSpace, ElExp, ElBoil, ElLight, HtDHW, ElClComf, ElHP, HtVent, ClTot, ElLightOut, ElPlug, ElPV, ElHWH, ElEV, ElFan, ElPump, ElImp
8100	1903	15585	1250	DH	DH	8.8	HtDH, ElImp
8101	1954	11810	800	GSHP, EB	GSHP, EB	11.1	ElHP, HtHP, ElImp, ElBoil
8102	2009	15127	1100	DH	DH	3.8	HtDH, ElImp, HtTot, ElExp, ElPV
8103	1916	7500	420	DH	DH	7.8	HtTot, HtDH, ElImp
8104	2016	9750	513	DH	DH	4.8	HtDH, ElFan, HtDHW, HtTot, ElImp, HtVent
8105	2011	8415	460	DH	DH	11.1	HtTot, HtDH, ElImp
8106	1900	5734	370	DH	DH	11.1	HtTot, HtDH, ElImp
8107	1982	6063	310	DH, HWH	DH	11.1	HtTot, HtDH, ElImp
8108	2009	7004	350	DH	DH	8.8	HtTot, HtDH, ElImp
8109	2018	10867	640	GSHP, HWH	GSHP, EB	5.2	HtSpace, ElFan, ElHP, ElLight, HtDHW, ElImp, HtSnow, HtTot, ElBoil, ElHWH
8110	1999	7081	440	DH	DH	11.1	HtTot, HtDH, ElImp
8111	1888	9604	425	EB	EB	4.1	ElBoil, ElImp
8112	1975	7287	660	EB, GSHP	EB, GSHP	9.9	ElBoil, ElImp
8113	1995	5690	360	DH	DH	9.7	HtDH, HtTot, ElImp, ElExp, ElPV
8114	1954	17589	1300	DH	DH	11.1	HtTot, HtDH, ElImp
8115	2016	9215	840	GSHP, HWH	GSHP, EB	5.7	HtHP, ElHP, ElImp, ElBoil, ElHWH
8116	1959	13408	840	DH	DH	11.1	HtTot, HtDH, ElImp
8117	1968	7480	1150	GSHP, EB, HWH	GSHP, EB	3.7	HtHP, ElHP, ClComf, ElImp, HtTot, ElBoil, ElHWH
8118	1913	7471	530	EB	EB	11.1	ElBoil, ElImp
8119	1954	8742	360	DH	DH	11.1	HtTot, HtDH, ElImp
8120	2010	13339	940	DH	DH	5.3	ElImp, HtDH, HtTot
8121	1873	4006	325	DH	DH	8.8	ElImp, HtDH, HtTot
8122	1953	8669	470	DH	DH	9.8	HtTot, HtDH, ElImp
8123	2010	11304	770	GSHP, EB, HWH	GSHP, EB	4.8	HtSpace, ElHP, ElImp, ElBoil, ElHWH
8124	1861	10724	530	DH	DH	1.2	HtTot, HtDH, ElImp
8125	1936	8695	700	DH	DH	1.2	HtTot, HtDH, ElImp
8126	1997	9234	720	DH	DH	11.1	HtTot, HtDH, HtDHW, ElImp
8127	2020		576	GSHP, HWH	GSHP, EB	2.8	ElEV, HtHP, ElHWH, ElHP, ElImp, HtTot, ElExp, ElBoil, ElPV
8128	1939	9736	720	DH	DH	8.7	HtTot, HtDH, ElImp
8129	1981	4723	330	GSHP, EB	GSHP, EB	8.5	HtSpace, HtHP, ElHP, ElImp, ElBoil
8130	1963	7914	540	EH	DH, EH	8.8	HtDH, ElImp
8131	1988	4706	372	DH	DH	4.1	HtTot, HtDH, ElImp
8132	2009	11373	685	DH	DH	11.1	HtTot, HtDH, HtDHW, ElImp
8133	1882	10075	250	DH	DH	5.7	HtTot, HtDH, ElImp
8134	1926	6540	490	GSHP, EB	GSHP, EB	4.8	HtHP, ElHP, ElImp, ElBoil
8135	2015	10150	780	DH	DH	5.5	HtTot, HtDH, HtDHW, ElImp
8136	1895	10549	690	GSHP, EB	GSHP, EB	5	HtHP, ElBoil, ElImp, ElHP
8137	1955	12453	720	DH	DH	5.3	HtTot, HtDH, ElImp
8138	1979	8631	400	EB	ASHP, EB	4.2	ElImp, ElBoil

The schools managed by Oslobygg use a variety of heating systems, but all have central heating systems that supply all or most of the building’s heat. Several schools are connected to Oslo’s district heating grid, using district heating as the primary source for both space heating and domestic hot water. Some of these buildings may also have supplementary electric hot water heaters in certain areas and/or electric heating coils for ventilation systems. Schools not connected to the district heating grid rely on electricity for heating. The most common setup among these is a combination of ground source heat pumps and electric boilers, which together cover both space heating and hot water demand. Generally, heat pumps are used as the primary heating source while the electric boilers or bio boilers are secondary heating sources that provide heating in periods with high demand or as back up heating if the heat pump fails. A few schools have alternative configurations, such as air-to-water heat pumps combined with solar thermal collectors and electric boilers, or ground source heat pumps paired with bio boilers instead of electric ones. Some schools rely solely on electric boilers to meet their entire heating demand.

## 4 Experimental Design, Materials and Methods

### 4.1 Source of data

The energy use of all public buildings in Oslo is monitored in Energinet which is an administration software tool for energy, waste and environmental monitoring: https://kiona.com/products/energinet . *Energinet is a software for energy, waste and environmental reporting, where energy meter data is directly imported via SFTP, email, industry protocols (e.g. OBIX), third-party APIs, and directly through Energinet’s API solutions.* Oslobygg monitors the energy use of several hundred buildings within Energinet, including schools, nursing homes and kindergartens. The types and number of energy meters as well as other meters (including water meters) varies a lot from building to building.

The raw data for the dataset presented in this data descriptor originates from the private Oslobygg account in Energinet (https://www.energinet.net/), collected in October-November 2023 and August 2024. The energy meters are connected to each building under Oslobygg’s Energinet profile. This includes the main electricity meters (Advanced Metering System, AMS-meters), whose calibration and accuracy are regulated by [10] and the main district heating meters, whose calibration and accuracy are regulated by [11]. Any additional sub-meters have been manually connected by the building owner (Oslobygg). The type, calibration, and accuracy of these sub-meters vary and are not documented. Some processing is performed within Energinet to convert measurements from the main and sub-meters from aggregated data into hourly energy use. In addition, Energinet applies interpolation to hours with missing data to prevent artificial spikes in the energy use caused by data gaps.

All other processing and post-processing of the data includes the selection of suitable buildings and meters to fit the study scope, relabelling of meters, unit conversions, combining weather data with energy use data, resetting timestamps to UTC, converting files to standard file formats, as well as simple data cleaning as described in the following section. These steps were performed using Python (v3.11) and the Pandas library (v2.2.3).

### 4.2 Data collection scope

The data collection aimed to assemble a historic dataset of hourly energy use in non-residential buildings with sub-meters for studying typical load profiles and developing methods for building classification and load disaggregation. Due to time constraints and data quality requirements, the scope was narrowed as follows:

• **Building type:** The dataset focused on schools, which represent a significant portion of public buildings and are typically equipped with multiple submeters and control systems.
• **Submetering:** Only schools with submeters for heating (e.g., electric boilers, heat pumps, or district heating) were included.
• **Data quality:** Buildings with accurate, real submeters for heating (not estimated or artificial data) were prioritized.

Out of 160 schools connected to Oslobygg Energinet, 58 met the first two criteria, and the raw data were extracted for these buildings. Later, 10 of these buildings were discarded due to previously undiscovered errors in the data, as described in the section below.

### 4.3 Data collection and relabelling of meters

**Fig. 7:**
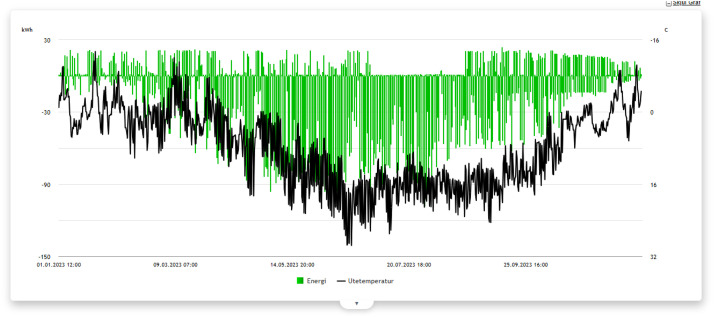
A “rest”-meter showing the difference between the main meter and sub-meters. The rest-meter is not a physical meter but calculated. The rest is sometimes negative, indicating measurement errors, double counting or local PV generation which is not measures by separate meters.

**Fig. 8:**
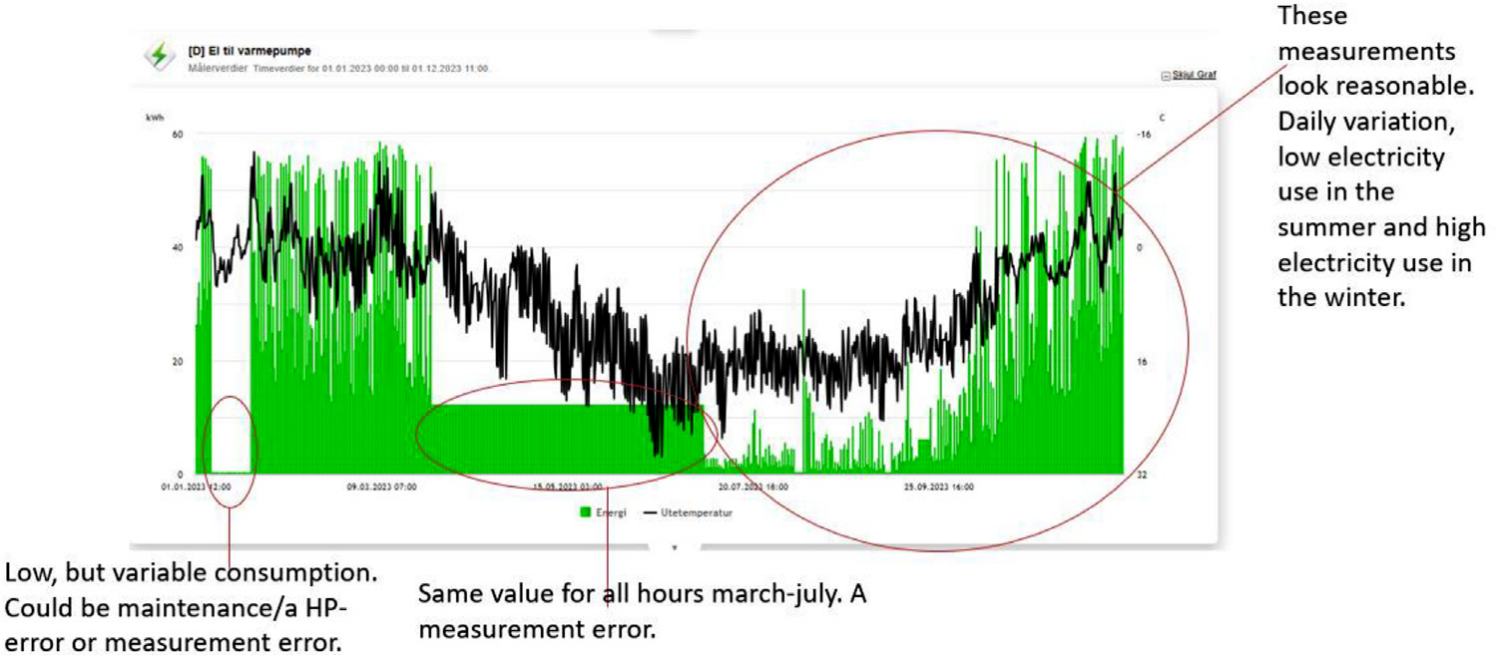
A measurement series of the heat pump (HP) in one of the buildings. The measurements have errors in parts of the time series, while other parts of the time series contain useful data.

For each school, the aim of the data collection was to collect energy use measurements, including main meters and sub-meters of energy used in the building and connect this to weather data at the building’s location. Only energy meters measuring end use of energy use in Wh or kWh data with sufficient quality was included. No indoor air parameter meters, water meters or flow meters were included. A visual inspection was carried out for each school to confirm the presence of at least one main meter (AMS) and a reliable heating meter (electricity or district heating). These meters needed to cover most of the building's heating demand and provide high-quality data with minimal gaps or many measurement errors (eg. Sub-meters with higher values than main meters, obvious outliers, negative “rest”-meters. The inspection was done manually by reviewing each building’s meters in Oslobygg Energinet and documenting their how well they fit with the scope). Typical measurement errors discovered through visual inspection is described in Fig. 8. This process ensured quality control by identifying issues such as missing data or overlapping readings. Common problems found during the inspection are shown in and . Typical errors include “rest”-meters showing negative values, indicating an overlap between meters or double counting of sub-loads. Other issues include missing datapoints, repeating values, and errors in the labelling of meters. A rest-meter with errors is shown in Fig. 7.

**Fig. 9:**
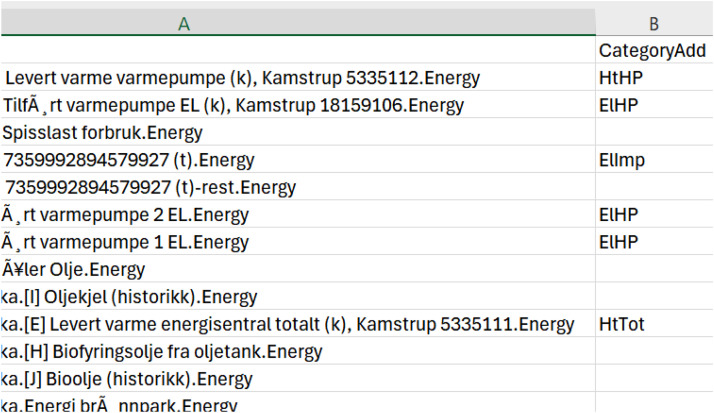
Relabelling of the meters for one of the schools

After selecting suitable buildings from Energinet that met the data quality requirements/scope, all meter data from these schools were downloaded in their raw form. Meters with sufficient data quality were retained and relabelled according to one of the meter names listed in Table 3, as shown in the example in Fig. 9. During relabelling, some meters were assigned to multiple columns. For example, the district heating main meter was added to both “HtTot” (total heating) and “HtDH” (total imported heating). If a building had multiple AMS meters, all were included in the “ElImp” (imported electricity) column. E.g. in some cases, the electric boiler was metered by a separate AMS meter, and so it was added to both “ElImp” and “ElBoil.” Similarly, if a building had multiple heat pump meters, they were combined into the “ElHP” category in the final dataset. shows an example of the original names of the meters of one of the buildings and which category they were added to in the final csv-file of the building.

### 4.4 Weather data

Each building file includes time series data for both weather and energy use. Meteorological weather data has been obtained for the geographical locations of the buildings from the MET Nordic dataset provided by the Norwegian Meteorological Institute. The MET Nordic dataset comprises post-processed products that describe both current and historical weather conditions. These products combine output from the MetCoOp Ensemble Prediction System (MEPS) with measurements from various observational sources, including crowdsourced weather stations. The resulting products are deterministic, meaning they provide only a single realization of the weather [12].

The building locations are identified using zip codes, and the geographical coordinates are then sourced from a database created by Erik Bolstad (https://www.erikbolstad.no/postnummer-koordinatar/).

Table 5 presents the collected weather data, listing both the dataset names and their corresponding names in the MET Nordic dataset.

**Table 5:** Weather data parameters and corresponding name in source database.

Name	Explanation	Unit	MET Nordic name	Comment
Tout	Temperature Outdoor	C	air_temperature_2m	-
SolGlob	Global Solar Horizontal Radiation	W/m^2^	integral_of_surface_downwelling_ shortwave_flux_in_air_wrt_time	Shifted backwards by one hour to align with “left label”.
WindSpd	Wind speed	m/s	wind_speed_10m	-
WindDir	Wind direction	deg	wind_direction_10m	-
RH	Relative humidity	%	relative_humidity_2m	

### 4.5 Cleaning of data

**Fig. 10:**
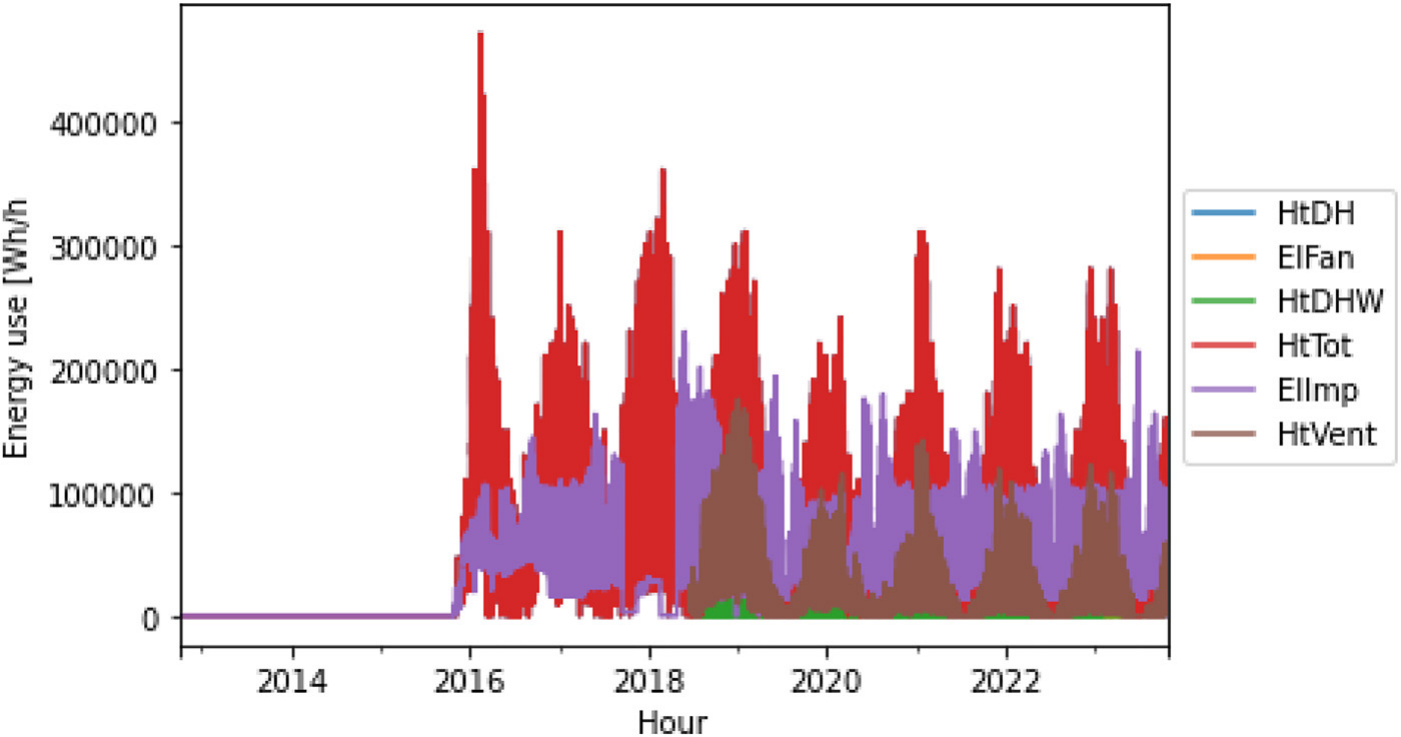
Time series before cleaning.

After selecting the eligible buildings and meters, relabelling the meters, and adding weather data, the time series data was cleaned. As previously noted, some meters provide high-quality data for certain periods but poor data for others. The first step in the cleaning process was to remove time periods with unreliable data (Fig. 10).

Additionally, the time series energy data was cleaned according to the following rules:

- Values exceeding 10 times the standard deviation plus the mean were considered outliers and set to NaN.
- All energy columns were standardized to Wh/h manually
- Negative values in energy columns (except for exported values) were set to NaN.
- Consecutive identical values appearing 23 times or more in a row were flagged as anomalies, and the corresponding days were removed by setting the values to NaN.

In this data descriptor we publish a lightly cleaned dataset rather than raw data. The cleaning process, conducted in consultation with the data owners, removed only evident errors (e.g., outliers and false zero values) to reduce the risk of misinterpretation. Additionally, the raw meter identifiers lacked a standardized naming convention and contained information which could be used for building identification, and the raw meters had various measurement units.

### 4.6 Meta Data

In addition to the time series data, contextual data about the buildings and their heating system, here referred to as meta data, has been collected for each of the selected school buildings. This collection process involved utilizing multiple sources and required considerable manual effort.

Several challenges were encountered during the meta data collection. The process necessitated careful consideration to resolve discrepancies across different data sources. For instance, the reported floor area of a school often varied between the cadastral register, the building's energy label, and given in Energinet. Resolving these inconsistencies required informed estimation to determine a reasonable floor area value. The full link of the websites accessed during the procurement of meta data is not given due to the need anonymize the buildings.

**Table:** 

Resource	Data	Note	Website
Energy Label of the building	Area Year of construction Heating source	The energy label contains information about whether the building has a heat pump or not, but not what kind (A2A, GSHP or ASHP), and if it has direct electric heating, but with any specifications.	https://portal.ems.enova.no/sok The energy label can also be found within Energinet for most buildings.
Ground bore holes database «Granada»	Boreholes (indicator of GSHP)	Grenada is a map of boreholes. To find out if a school with heat pump has a ground source or air source heat pump, the school is studied on the map to look for boreholes.	https://geo.ngu.no/kart/granada_mobil/
School websites	Number of students/ Year of construction Postal code	The year of construction in the meta data is generally set to the year the building first opened, unless, the school has been completely rebuilt.	https://aktuelt.osloskolen.no/
Energinet	Area Heating source Year of construction	A lot of information about the schools will also be available in Energinet, such as the area and year of construction. Heating source may not be explicitly given, but available meters may tell what kind of heating is available.	https://www.energinet.net/
Doffin (Database for public procurement)	Description of technical installations	Procurements of schools technical installations and renovations can give some technical insight which can be useful such as PV capacity of installed system.	https://www.doffin.no/
Local websites and history sites	Construction year History Number of students Renovations Technical systems	There are several local history web pages about the schools and the buildings.	https://oslobyleksikon.no/ https://kulturpunkt.org/ https://digitaltmuseum.no/ https://oslobyleksikon.no/

## Limitations

Users should exercise caution when down sampling the dataset, due to potential missing data points. It is important to assess the data quality of each meter before resampling to ensure reliable analysis.

Data from the AMS meters and district heating meters are generally considered reliable. These meters are designed to meet strict industry regulations, ensuring precise energy use or production measurements for billing purposes. In contrast, while sub-meter data remains valuable, it may require additional validation before being used in further research or analysis. The reliability of sub-meter measurements can vary [13], making it essential to evaluate their quality on a case-by-case basis. In some cases, certain periods of sub-meter measurements may need to be discarded if found unreliable or affected by measurement errors.

The school buildings in the dataset are all located within Oslo municipality, placing them in the same climate zone. Additionally, being in an urban area, a significant proportion of these buildings are connected to the district heating grid. The buildings are all managed by the same owner, Oslobygg KF. Users should take these factors into account when interpreting the data and exercise caution when generalizing the data of these schools to the entirety of Norwegian school buildings.

## Ethics Statement

The authors have read and follow the *ethical requirements* for publication in Data in Brief and confirming that the current work does not involve human subjects, animal experiments, or any data collected from social media platforms.

## Credit Author Statement

For this data descriptor:

**Synne Krekling Lien:** Data Curation, Validation, Writing - Original Draft, Software, Writing - Review & Editing. **Bjørn Ludvigsen:** Data Curation, Software, Writing - Original Draft. **Harald Taxt Walnum:** Data Curation, Writing - Original Draft**., Aileen Yang**: Writing - Review & Editing**. Åse Lekang Sørensen**: Project administration, Writing Review and editing, **Kamilla Heimar Johra:** Writing - Review & Editing, Project administration.

For the related research article:

**Synne Krekling Lien:** Writing – review & editing, Writing – original draft, Visualization, Validation, Software, Methodology, Investigation, Formal analysis, Data curation, Conceptualization. **Ada Canaydin:** Writing – review & editing, Writing – original draft, Visualization, Methodology, Investigation. **Clayton Miller:** Writing – review & editing, Visualization. **Chun Fu:** Writing – review & editing. **Hussain Kazmi:** Writing – review & editing. **Jayaprakash Rajasekharan:** Writing – review & editing, Supervision.

## Data Availability

data.sintef.noCOFACTOR-SBHUB Oslo: Hourly Sub-Metered Energy Use Data from 48 public School Buildings in Oslo, Norway (Original data).

## Appendix Supplementary materials

Image, application 1
