# Supplementary material for: COFACTOR-SBHUB Oslo: Hourly Sub-Metered Energy Use Data from 48 public School Buildings in Oslo, Norway
Source: Data Brief. 2025 Nov 15;63:112288. doi: 10.1016/j.dib.2025.112288 (PMC12703956; doi:10.1016/j.dib.2025.112288)
Supplement: Supplementary file 1 [file mmc1.docx]

Cross-domain disaggregation of electricity for heating in all-electric school buildings –learning from school buildings with district heating

Synne Krekling Lien^1,2^
Ada Canaydin^3^
Clayton Miller^4^
Chun Fu^5^Hussain Kazmi^3^
Jayaprakash Rajasekharan^1^

*Corresponding author: synne.k.lien@ntnu.no

1. Department for Electric Energy, Norwegian University of Science and Technology (NTNU), NO-7491 Trondheim, Norway
2. SINTEF Community Oslo*,* NO-0314, Oslo, Norway
3. Department of Electrical Engineering (ELECTA-ESAT), KU Leuven, BE-3001, Leuven-Heverlee, Belgium
4. Department of the Built Environment, National University of Singapore, 119007
5. Centrica Business Solutions, BE-2600, Antwerpen, Belgium

**Keywords**

Energy use, buildings, disaggregation, machine learning, AMS-data, district heating, transfer learning, hourly

**Highlights**

- Most buildings with electric heating lack sub-meters for heating loads.
- Disaggregation of electricity for heating from hourly AMS meters in buildings.
- Cross-domain training on energy measurements from buildings with district heating.
- Data from 74 school buildings including novel dataset.
- CatBoost achieves 0.91 R², 2.6 % NMAE and 8 % peak load error for disaggregation.

Abstract

Electric heating is widespread in Norwegian buildings and significantly contributes to peak loads in the electricity grid. Non-residential buildings are typically heated either by district heating or a combination of electrical heating appliances. Despite its widespread use, most buildings lack sub-meters for electric heating. As a result, the true potential for energy efficiency and load flexibility from heating appliances in buildings remains unknown. Non-intrusive load monitoring and disaggregation techniques offer alternatives to sub-metering by using data-driven methods to extract electricity use for appliances from time-series data. However, little research has been conducted on disaggregating electrical heating loads from low-resolution data, partly due to the scarcity of sub-metered training datasets. Unlike all-electric buildings (EHBs), district heating buildings (DHBs) typically have separate, hourly heating energy meters. This paper examines feature extraction and multiple machine learning algorithms for disaggregation of electricity for heating from AMS-meter data in EHBs, and how cross-domain training from DHBs can contribute to this task. We use sub-metered data from 74 school buildings (54 DHBs and 20 EHBs) in Norway with over 3.8 million hours of recorded measurements, where parts of the dataset are published in a novel public dataset. Results show that CatBoost achieves high performance in disaggregating electricity for heating in EHBs when trained on data from DHBs, with an R² value of 0.91, NMAE of 2.6%, and a peak load estimation error of 8%, which is an improvement compared to training on EHBs. The study also shows that feature engineering can improve the disaggregation performance in some, but not all EHBs.

1. **Introduction**

### Background

Buildings account for roughly a third of end energy use and emissions in many countries across the world. More efficient end use is therefore seen as key in climate change mitigation efforts[1]. Data availability is central to this in several ways. First, electricity usage data can be analysed to provide insights and feedback to building occupants to help them reduce their demand, emissions or costs[2]. Second, these insights can be used to optimize electricity usage via algorithmic means [3]. Third, these operational insights can also be leveraged to optimize future design choices[4].

However, despite significant progress in data gathering efforts, there remain large gaps in the available data on how buildings consume electricity[5]. This is driven by uneven adoption of advanced metering infrastructure, where installation rates still vary greatly by country and region (e.g. while Norway has almost universal coverage, Germany lags far behind[6]). The reasons for this heterogeneity are manifold, but even in regions with near-universal coverage, metering infrastructure often brings only surface-level insights. More specifically, smart meters in their current form do not provide any insight into behind-the-meter electricity usage, yet this level of detail is arguably essential for enabling the smart energy management. Installing sub-meters, which measure electricity demand for each appliance individually, is however economically and technically infeasible in most real-world conditions.

Non-intrusive load monitoring (NILM), or energy disaggregation, has emerged as a popular alternative to sub-metering in recent years[7]. Disaggregation algorithms essentially decompose the electricity demand time series into different appliance loads, which can then be used to optimize specific loads or provide human users with feedback and insight. These include methods which utilize unsupervised techniques, e.g. Additive Factorial Hidden Markov Model (AFHMM) which was used to disaggregate the REDD dataset (1 Hz resolution) for several appliances, including an electric furnace [8]) as well as supervised learning algorithms e.g., sparse-coding which was used for disaggregation of various appliances on a private dataset with hourly loads from 590 European households, including heating and cooling appliances [9]. Supervised learning with linear regression serves as a simple baseline for disaggregation and has been effective in some disaggregation tasks [10], [11], [12]. Random Forest Regression is also widely used for supervised disaggregation as it handles non-linear relationships better [13], [14]. CatBoost is another model which leverages gradient boosting and is efficient for non-normalized data. LSTM has been widely employed for disaggregation tasks due to it being as it is well-suited for capturing temporal dependencies [15], [16], [17], [18], [19].

However, these algorithms suffer from many shortcomings of their own, both in terms of data requirements as well as model validation and operationalization. More concretely, they often assume the availability of ground truth data (i.e., which appliances were in use at what time), often at very high sampling rates i.e., at up to tens of thousands of times per second. These high sampling rates can help identify the electrical 'signature' of different appliances. Furthermore, there is a notable gap in existing literature on the disaggregation of electricity for heating from hourly smart meter data, and especially for buildings with more than one heating technology [20]. Heating demand is important to model accurately, because in colder climates it represents the largest draw in buildings and the main contributor to the peak load in buildings. It also provides the bulk of available energy flexibility in such cases. One reason for a lack of focus on heating disaggregation may be the lack of available data for training. In fact, in most countries (including Norway, which is the focus of this study), there are currently no regulations requiring sub-meters for electric heating appliances, and as a result, there are few buildings with sub-meters for electric heating appliances (e.g., electric boilers, heat pumps, floor heating, panel heaters, etc.) and so testing and validating methods for disaggregation of electricity for heating remains challenging. Buildings with a district heating connections however typically have a main meter for district heating in addition to the advanced metering systems (AMS meter). Due to this, a question arises: can measurement data from buildings with district heating (DHB) be used to train a supervised model to disaggregate electricity for heating in all-electric buildings (EHB) in a transfer learning or cross domain setting?

Transfer learning is a supervised machine learning technique where a model, often a neural network, is trained on one problem or dataset (referred to as the source) and is then adapted to a related task (referred to as the target) to improve predictive performance. This is especially relevant when data in the target domain is scarce, but the source has abundant data (either due to simulations or observations in similar contexts). Different applications of transfer learning can already be found in energy time series prediction, estimation, and forecasting. For instance, [21] used transfer learning to improve photovoltaic (PV) energy generation forecast models by pre-training on simulated data and then fine-tuning on observational data. Likewise, [22] used transfer learning to improve and automate the learning process of a black box of hot water system models, using real-world data from 61 houses with two different types of hot water systems. In a similar vein, [23] investigated how transfer learning could be used to enhance building energy consumption forecasting with transformer architectures. In all these cases, transfer learning significantly improved the results of the models with less training data and showed promising results for applications in building modelling and simulations.

Transfer learning has been explored for energy load disaggregation in several studies. When using transfer learning, a pre-trained model (typically a neural network) is adapted to a new domain by fine-tuning or retraining it on new data. Some model frameworks, such as gradient boosting frameworks, are not inherently designed for transfer learning in the way neural networks are since they operate on tabular data. Thus, cross-domain application relies on the direct use of a model in a new domain without any modification (also referred to as zero-shot transfer), assuming that the patterns learned during training are robust enough to generalize across domains. Transfer learning for disaggregation has been investigated in [24] by training Seq2point models on 8-second data from REFIT[25] and testing on REDD[26] and UK-DALE[27], finding that CNN layers trained on washing machines could generalize to other appliances. [28] examined transfer learning with sparse coding models, testing domain shifts within REFIT (for different household sizes in train and test) and between training on REFIT and testing on IRISE. Results showed performance comparable to regular models, though both struggled with the significant domain shifts from one dataset to another. [29] applied a sparse coding approach with a deep temporal model (LSTM-AE) on REDD (1-second resolution) but focused on disaggregating appliances within the same dataset, where the domain shift was small and only from some houses to other houses. Similarly, [15] used semi-supervised deep transfer learning (1D-CNN with a regressor) to disaggregate electricity for EVs, dryers, refrigerators, and furnaces across three houses from Pecan Street, again only having a domain shift from some houses to other similar houses, and While “domain adaptation” improved results, performance for furnaces remained poor.

### Motivation

Traditionally, Norway has had the lowest electricity prices in all of Europe due to an abundance of hydropower [30]. As a result, more than 80 % of the end-use in households and more than 70 % of the end-use in non-residential buildings comes from electricity, compared to a much lower share of 25 % in the EU [31]. The main explanation is that electricity is used to cover a large share of the heating demand in buildings, either by use of electric boilers, electric floor heating, electric panel heaters, and/or heat pumps. A main motivation for disaggregating heating loads from the AMS-meters of buildings is the heavy reliance on electricity to meet heating needs leads to high grid peaks and large variations during winters (and extreme cold waves) [32]: the observed peak load of Norway’s electricity use in 2021 reached 25.2 GWh/h (at 9 AM on February 2^nd^), while the average hourly load was 15 GWh/h [33]. As the transportation and industrial sectors become increasingly electrified, this is expected to rise further, especially during peak hours, necessitating substantial investments in the grid [34]. One cost-effective alternative to this grid expansion is to implement strategies for peak reduction and demand-side flexibility [35], [36], which can be achieved by managing building electricity consumption more efficiently during peak periods. To unlock this potential, there is a need for greater insights into electricity consumption and how different loads contribute to the hourly load seen by the buildings’ main meter.

### Research gap

Even though transfer learning has shown promise for certain disaggregation tasks, it has not been tested on low-resolution data for disaggregating heating loads. Due to the limited availability of all-electric buildings (EHB) equipped with sub-metering for electric heating, there is considerable potential in applying supervised cross-domain adaptation or transfer learning from district heating buildings (DHB) to disaggregate heating-related electricity consumption from hourly AMS-meter data in EHBs. However, this approach remains largely unexplored, representing a clear research gap. This study aims to address this gap by investigating whether such models can be effectively applied to EHBs to estimate heating-related electricity use. If successful, the methodology could be extended to other building categories, enabling the disaggregation of heating energy without the need for extensive sub-metered data collection.

### Contributions

This article is a case study on disaggregating electricity demand in school buildings in Norway and investigates the research question: “How can we best disaggregate electricity for heating in all-electric buildings using data-driven methods and can data from district heating buildings help us reach this target?”. To answer this question, a study is conducted on a dataset with several years of hourly data from 74 school buildings in Norway, comprising 54 DHBs and 20 EHBs with sub-meters for heating collected from 3 locations in Norway, consisting of almost 4 million total hours of energy use data. The paper makes the following important contributions:

1. The paper presents new typical daily load curves, ET curves and energy signature curves for EHB and DHB school buildings in Norway and shows examples on how these can be used as features for heating disaggregation.
2. The paper develops a cross-domain, zero-shot TL-based algorithm to disaggregate electricity use for heating from AMS-meters in all-electric school buildings, trained on metered data from district heating buildings.
3. The method is thoroughly validated using sub-meter data from schools with electric heating, analyzing performance at the individual building level to assess how it varies with different building configurations. Additionally, the study explores feature extraction, feature importance and model transferability analysis using t-SNE to better understand the key drivers of the model’s performance.

A dataset containing hourly energy use measurement data between 1-11 years of data from Oslo municipality is also published together with this paper in [37].

# Data description and processing

## Dataset

This article is a case study that focuses exclusively on school buildings to explore the cross-domain disaggregation approach within a single building category. Here, buildings are categorized as either district heating buildings (DHBs) or electric heating buildings (EHBs) which are sometimes also referred to as all-electric buildings.

In *DHBs*, the buildings are connected to both a district heating network and the electricity grid. District heating energy is used for space heating and domestic hot water heating, with total heating energy (Y) measured by a district heating meter. Electricity is used for all other purposes, such as lighting, equipment, ventilation, and, in some cases, cooling. The electricity consumption is measured through the AMS meter. The total imported energy (X) in DHBs is the sum of imported electricity and imported district heating energy.

In *EHBs*, all imported energy comes from electricity, which is measured through the AMS meter. These buildings are also sometimes referred to as “all-electric buildings”. Therefore, the total imported energy (X), is equal to the imported electricity. To measure the electricity/energy used specifically for heating (Y), sub-meters are required. However, most EHBs do not have sub-meters installed, making direct measurement of heating electricity challenging. In EHB schools, heating is typically provided using electric boilers, sometimes in combination with heat pumps and/or other heating appliances. The delivered energy for heating in EHBs (Y) hence becomes the sum of electricity for the heat pump and the electricity for the electric boiler.


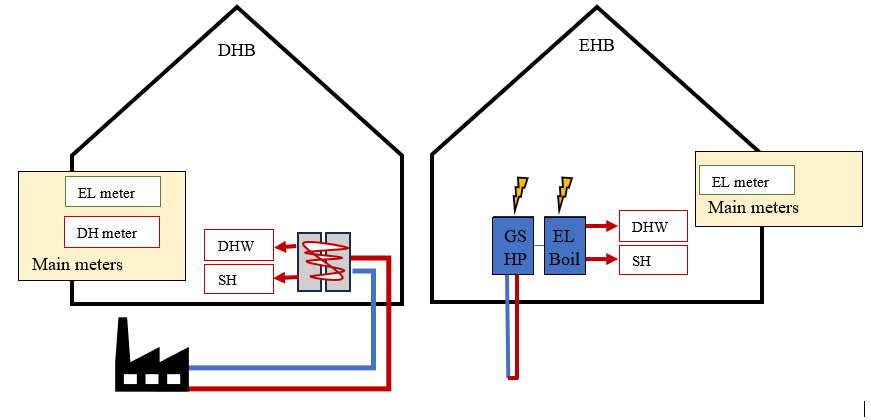


Figure 1 Description of heating system in DHBs and EHBs. Abbreviations include “DHW”: “Domestic Hot Water heating”, “SH”: “Space Heating”, GSHP = “Ground Source Heat Pump”, “ElBoil”: Electric Boiler.

This study analyses a dataset of 74 school buildings from three locations - Oslo, Drammen, and Trondheim - collected from three different datasets. An overview of the buildings in the dataset, categorized by heating type and location/reference is presented in Table 1. To validate the disaggregation, only buildings with existing sub-meters for heating are included in the dataset. The Drammen buildings are from [38], a public dataset containing four years (2018-2021) of hourly energy and climate data from 45 public buildings. This dataset includes 6 schools with sub-meters for heating, all classified as EHBs, which are all included in this study. The Trondheim buildings come from [39] which provides hourly data from 77 buildings owned by Trondheim municipality, covering the period 2018–2022. The Trondheim dataset includes 31 school buildings with sub-meters, all classified as DHBs, which are included in this study. Additionally, a novel dataset of hourly energy time series from 48 schools in Oslo is published with this article in [40]. Of these, 37 buildings are included in this study, while the rest were excluded due to either an inability to be categorized as EHBs or DHBs (e.g., using bio-boilers), had local energy generation without sufficient meters, or had insufficient sub-metering for heating.

Table 1 Overview of buildings per building category, location, and heating category (DHB/EHB).

| *Number of buildings per location* | *Number of DHBs* | *Number of EHBs* | *Total* |
| --- | --- | --- | --- |
| Drammen [41] | 0 | 6 | 6 |
| Oslo [40] | 23 | 14 | 37 |
| Trondheim [39] | 31 | 0 | 31 |
| Grand Total | 54 | 20 | 74 |

The combined dataset consists of one file per building, all following a consistent format. Each file contains metadata and hourly time series data, including weather parameters and energy meter readings. For a detailed description of the dataset structure, refer to [40]. An overview of the data period of each building in the dataset is shown in Figure 2. In this visualization, each row corresponds to a building, and each column represents a single time step, with black spots or streaks indicating missing data.


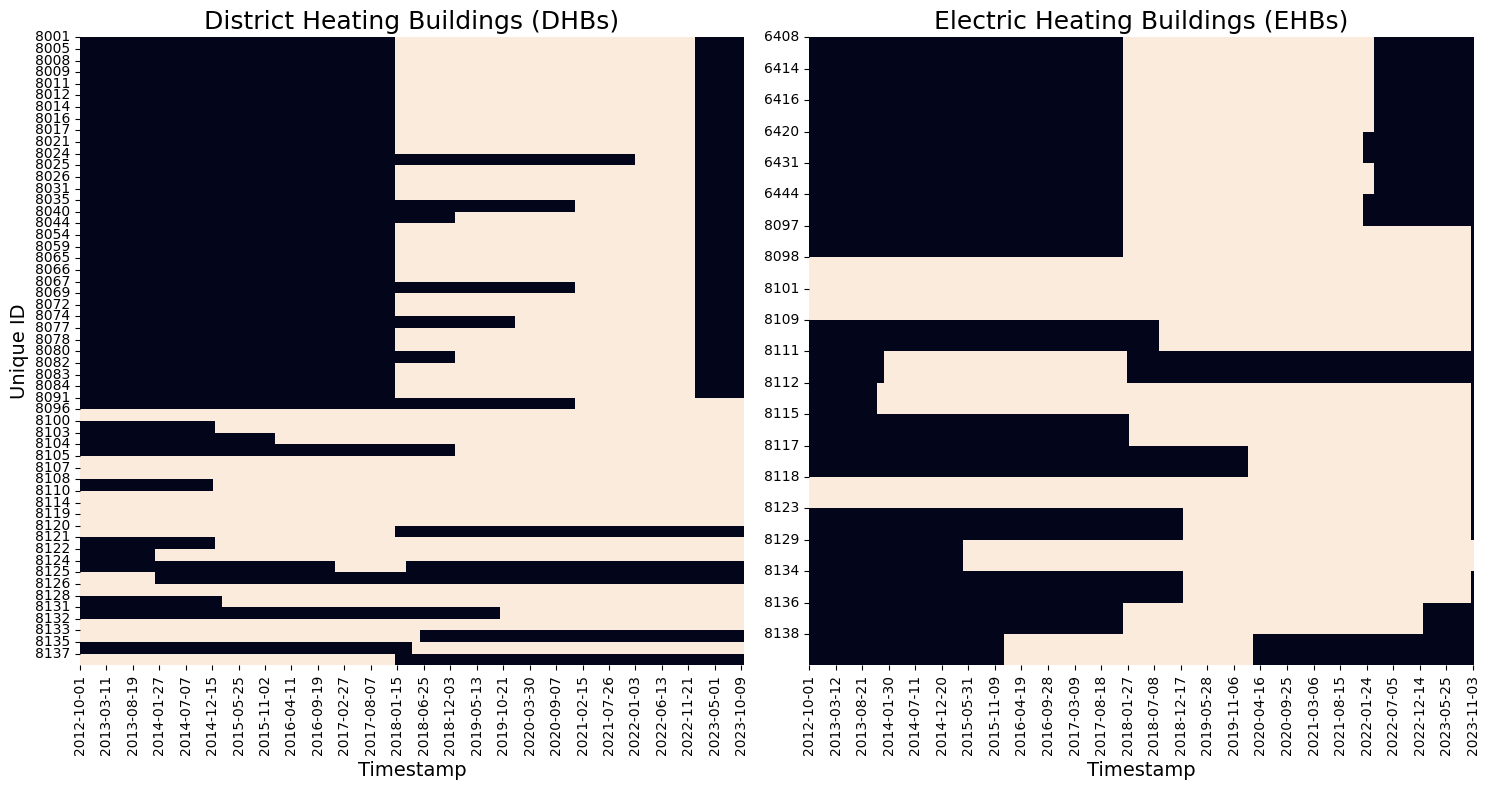


Figure 2 Data duration per building in the dataset. Black = Duration with no available data.

## Treatment of data

### Pre-treatment and Train/test split

To standardize the treatment of EHBs and DHBs, a new column, "X," is added to all building files, representing the total energy use of each building. The target variable, "Y," is introduced as the total energy used for heating. All other energy meters, including energy sub-meters and main meters, are removed from the files. In some model approaches, the disaggregation target is set as Y/X instead of Y, representing the share of energy used for heating - a value between 0 and 1. Table 2 explains which meters contribute to total energy (X) and heating energy (Y) for EHBs and DHBs respectively.

This study investigates a data-driven approach to disaggregation. To do this, the building files are sorted into train, test and validation sets. EHBs and DHBs are treated separately, with buildings in each group randomly assigned to train/test/validation as shown in Table 2. For DHBs, approximately 75% of buildings are allocated to training, with the remaining 25% split between validation and test sets. For EHBs, due to their smaller sample size, 30% are assigned to testing for a more comprehensive performance assessment across different buildings, while 10% go to validation and 60% to training. Unlike the common approach of splitting by time for time-series prediction and disaggregation, the dataset is divided by buildings to minimize the risk of bias and overfitting during training. This approach ensures that the method can generalize to previously unseen buildings.

Table 2 Overview of the buildings in each set and the number of buildings

| *Cat.* | *Set* | *Number of*  *Schools* | *N hours* | *Total years of data in set (buildings combined)* | *Average N years of data per school in set* | *Total energy (X)* | *Energy use for heating,*  *Load to disaggregate/*  *estimate (Y)* | *Alternative target: Share of energy used for heating (Y/X)* |
| --- | --- | --- | --- | --- | --- | --- | --- | --- |
| DHB | Train | 37 | 1 860 217 | 212 | 6 | DH + EL | DH | DH/(DH+EL) |
|  | Val | 8 | 460 852 | 53 | 7 |  |  |  |
|  | Test | 9 | 465 380 | 53 | 6 |  |  |  |
| EHB | Train | 12 | 599 523 | 68 | 6 | EL | ElBoil (+ ElHP if available) | (ElBoil + ElHP)/EL |
|  | Val | 2 | 72 339 | 8 | 4 |  |  |  |
|  | Test | 6 | 376 895 | 43 | 7 |  |  |  |

Figure 3 presents the load profile for the total imported energy (X) and the measured electricity for heating (Y) in one of the EHBs from the test set. The figure displays hourly values for both X and Y throughout the building’s data series, covering the period from 2015 to 2023 (nine years). The load profiles reveal significant seasonal variations in electricity consumption, which are strongly correlated with fluctuations in electricity used for heating. The load profiles for all the EHBs in the test set are included in Appendix A.


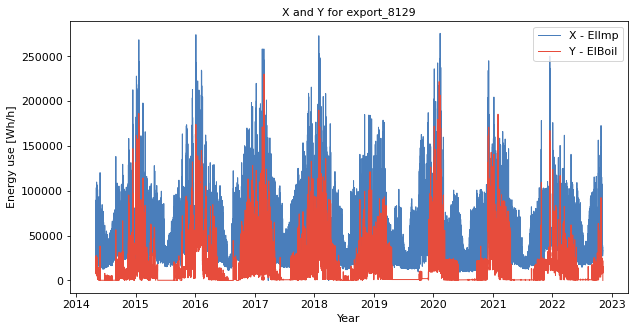


Figure 3 Load profile for one EHB School in Oslo (id 8129) built in 1981 with 4723 m2 area. The school uses a ground source heat pump and electric boiler for heating. “Y”, energy for heating, is the sum of electricity used in the heat pump (“ElHP”) and in the electric boiler (“ElBoil”).

# Methodology

To investigate how electricity use for heating can be disaggregated in all-electric buildings using data-driven methods, and whether data from district-heated buildings can support this goal, a dedicated data handling and methodological pipeline is followed, as illustrated in Figure 4. The pipeline includes pre-processing of data from he three locations, categorizing buildings as either all-electric (EHBs) or district-heated (DHBs), and further splitting them into training, validation, and test subsets. The results are presented in four main steps: 1) Analysis of energy use patterns in school buildings, 2) Model selection and target evaluation, 3) Building-level evaluation and 4) Feature engineering evaluation and exploration. Each step draws on different subsets of data from the pipeline, depending on its specific focus and requirements.


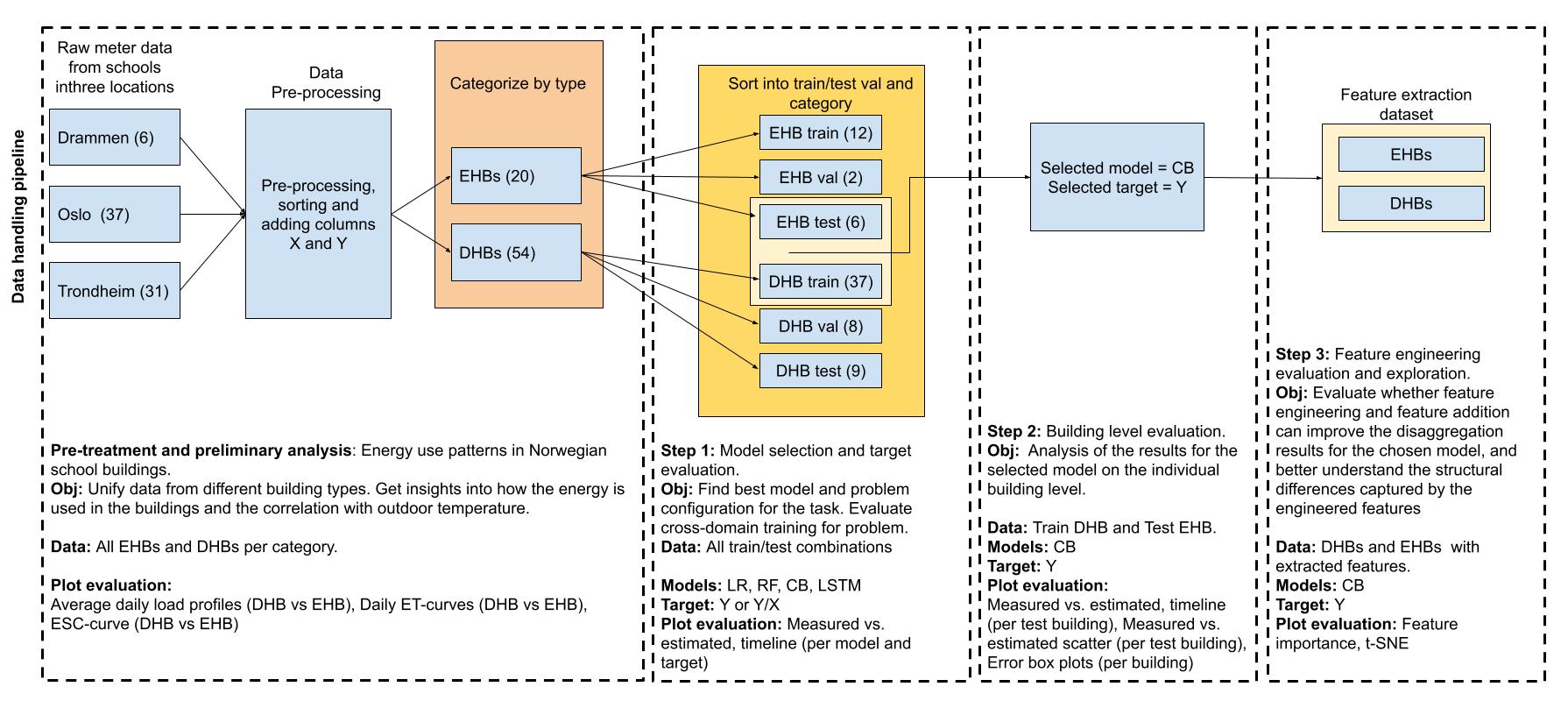


Figure 4 Data handling pipeline and methodology flow.

## Models and experiment setup

### Train/test combinations

This study explores the potential of using models trained on district-heating buildings (DHBs) to disaggregate hourly electricity consumption for heating in all-electric buildings (EHBs) based on their AMS-meter data. To assess this, various train/test set combinations are tested, as shown in Table 3. Training and testing are performed on both same-domain applications (e.g., EHB-train to EHB-test) and cross-domain applications (e.g., DHB-train to EHB-test), as well as on a combined training set of EHBs and DHBs. The same-domain sets are used as a baseline for the disaggregation, to see how well the trained model transfers to similar buildings. The DHB train/DHB test combination is simply a test, as the district heating energy in DHBs is always known in reality. The cross domain (DHB to EHB) is the main target, as if this proves to be efficient, it could be applied to other buildings were training data from EHB-buildings is scarce. The combined training set is used to examine whether increasing dataset size and incorporating greater variability by mixing both building types enhances accuracy.

Table 3 Train/test sets.

| *Train-*  *Test Combo* | *Train set* | *Validation set** | *Test set* |
| --- | --- | --- | --- |
| 1 | DHB Train | DHB Val | DHB Test |
| 2 |  |  | EHB Test |
| 3 | EHB Train | EHB Val | EHB Test |
| 4 | DHB Train + EHB Train | DHB Val + EHB Val | EHB Test |

*Validation set only used for training with LSTM model

### Machine learning models

This study evaluates four different machine learning models for the task of disaggregating hourly electricity use for heating: 1) *Linear Regression (LR), 2) Random Forrest Regression (RF) 3) CatBoost Regressor (CB)* and *3) Long Short Term Memory (LSTM).*
The model configurations are given in Table 4. The models are tested with the different train/test splits (Table 3) and target variables (Y and/or Y/X), except LSTM, which is only tested with Y/X as LSTM generally require normalized or standardized data to perform well. A normalized Y alone would not be meaningful, as disaggregation aims to estimate Y in relation to X.

| Model | Target | Target |
| --- | --- | --- |
| Linear regression | Model definition and settings: <https://scikit-learn.org/stable/modules/generated/sklearn.linear_model.LinearRegression.html>  Specification: LinearRegression() | Y |
|  |  | Y/X |
| Random Forrest  Regressor | Model definition and settings: <https://scikit-learn.org/stable/modules/generated/sklearn.ensemble.RandomForestRegressor.html>  Specification: RandomForestRegressor(random_state=42, n_estimators=100) | Y |
|  |  | Y/X |
| CatBoost Regressor | Model definition and settings: <https://catboost.ai/docs/en/concepts/python-reference_catboostregressor>  Specification: CatBoostRegressor(verbose=0, random_seed=42) | Y |
|  |  | Y/X |
| LSTM | Model definition and settings: <https://www.tensorflow.org/api_docs/python/tf/keras/layers/LSTM>  Specification: Two LSTM layers (64 and 32 units) followed by a dense layer for predictions. It uses the Adam optimizer with a 0.001 learning rate and mean squared error as the loss function. Early stopping prevents overfitting, and the learning rate adjusts dynamically when progress stalls. The model trains for up to 50 epochs with a batch size of 32. | Y/X |

Table 4 Model configurations.

## Feature engineering

The treated building files includes the features timestamp ("TimeStamp"), outdoor temperature ("Tout"), wind speed ("WindSpd"), wind direction ("WindDir"), Global Solar Horizontal Radiation ("SolGlob”) and total energy (“X”). As a part of the study, it is explored whether feature engineering can enhance model performance by including additional features, detailed in Table 5. The additional features from ‘Feature engineering’-category were selected because they are derived from the original features without requiring extra data collection if AMS measurements and location are known. To assess the impact of metadata on the disaggregation performance, a separate feature engineering analysis includes meta data features (floor area and year of construction), and additionally ET-curve parameters (energy-temperature relationships).

Table 5 Added features

| *Type* | *Feature category* | *Features* | *N* |
| --- | --- | --- | --- |
| Original | Original features | Tout, SolGlob, WindSpd, WindDir, X | 5 |
| Feature engineering | Datetime features | Features extracted from “TimeStamp”: Hour, day of week, season  Weekend, sin of hour, cos of hour | 6 |
|  | Daily features | Mean, Max, Min, Standard deviation (Std), Variance, Mean/max, Mean/Std, Mean/Var for X | 8 |
|  | Rolling window features (lag and lead) | Min X, Max X and Mean X for a rolling window set between +- 1 and +- 24 hours (both backwords and forwards) | 144 |
|  | Rolling window features time drop | Min X, Max X and Mean X for a rolling window set between -5 and -2 hours before current hour to detect ramp up | 5 |
|  | Lag and lead features | Lag/lead for X between +/- 1 to 12 between current X and X with lag/lead +/- 1-12 hours. | 48 |
|  | SAX features | The symbolic aggregate approximation (SAX) is a time series representation method [42]. SAX features in this function work by discretizing the time series data into symbolic letters based on calculated breakpoints, simplifying the data representation. Then, the function aggregates and counts the frequency of these symbols on a daily basis, enabling the analysis of daily patterns and their distribution in the time series data. 4/6//12/14 H and 6/8/12 | 12 |
| Additional feature analysis | ET-curve features | CPT, B0, B1, B2 for hourly ET-curve of each building. | 4 |
|  | Meta data features | Floor area, year of construction | 2 |

## Performance metrics and model evaluation

To evaluate the disaggregation performance, the performance metrics in Table 6 are used. Normalized Mean Absolute Error (NMAE) provides a scale-independent measure of error, making it useful for comparing buildings of different sizes. Peak Demand Error (PDE) assesses how well the model captures peak loads, which is important for evaluating the peak reduction and demand response potential in Norway. R-squared (R²) measures the proportion of variance explained by the model. The peak load error metric is rarely used in literature, but it is included in this analysis because electricity for heating in buildings is the primary dimensioning factor for the grid in Norway. Additionally, the peak power of a building can affect the grid tariff of costumers and is therefore closely related to demand response motivation during peak hours.

Table 6 Performance metrics.

| *Name* | *Short* | *Description* | *Formula* |
| --- | --- | --- | --- |
| Normalised Mean Absolute Error | NMAE | The mean absolute error divided by the mean of the observed values, providing a scale-independent assessment of prediction error. | $NMAE=\frac{\sum_{i=1}^{N} \left\vert y_{i}-\hat{y}_{i} \right\vert}{n\cdot\bar{y}}$ |
| Peak Demand Error | PDE | The error of the estimated peak load vs. the measured peak load. | $PDE= \frac{\left\vert max(y_{i})-max(\hat{y}_{i}) \right\vert}{max(y_{i})}$ |
| R-squared | R² | The coefficient of determination, quantifies the proportion of the variance in the dependent variable that is predictable from the independent variables in a regression model. | $R^{2}=1-\frac{\sum{{(y}_{i}-\hat{y}_{i})}^{2}}{\sum{{(y}_{i}-\bar{y})}^{2}}$ |
| $y_{i}$ true measurement values for point i $\hat{y}_{i}$ estimated value for point i $\bar{y}$ average values of all true measurement values $n$ number of datapoints | | | |

The disaggregation performance is evaluated at two levels:
*1) Full Dataset Evaluation*: This level is used for the model evaluation and feature extraction performance assessment. All the data from the buildings in the test sets are combined into one continuous dataset. This approach allows us to evaluate the overall performance of the disaggregation models across the entire dataset without focusing on individual building differences. It is important to note that the buildings in the dataset have varying time series lengths, meaning some buildings have more data points than others, making them have a greater weight in this evaluation level. The average performance is measured per hour in the test-sets.
*2) Per-Building Evaluation*: On this evaluation level, we evaluate the performance of the disaggregation method for the individual buildings in the test sets. This is only done for the selected model. Each building is evaluated separately, providing an understanding of which factors and patterns contribute to the success/failure of the disaggregation when new, unseen buildings are introduced. The average performance is measured per building.

## Preliminary analysis of Energy use patterns in Norwegian school buildings

This sub-section presents the results for daily load profiles and ET curves for EHB and DHB school buildings in Norway from all the 74 buildings in the dataset. The objective is to outline daily energy use patterns and temperature dependence for school buildings in Norway and give better insight into their energy consumption patterns.

Figure 5 and Figure 6 present the average specific (per m^2^) daily load profiles for X (total energy), Y (energy for heating), and imported electricity (“ElImp”) per square meter for the schools in the dataset, categorized into DHBs and EHBs. In the EHBs, ElImp is equal to X (blue curve). The figures indicate that the daily load profile of X in both EHBs and DHBs follows a similar pattern, with a morning peak likely due to the ramp-up of heating and ventilation systems followed by a gradual reduction in energy use throughout the school day and a consistently low energy demand during the night. Additionally, the load profiled of Y closely correlates with that of X in both building types. X and Y are higher in the DHBs compared to the EHBs which indicate a lower energy demand and/or higher efficiency for the heating systems in the EHBs due to the prevalent use of heat pumps.

| 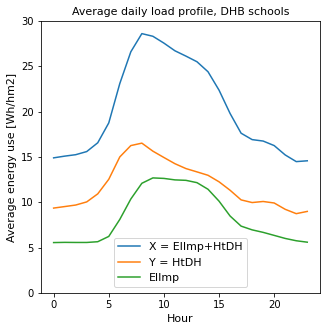  Figure 5 Average daily load profile for DHBs | 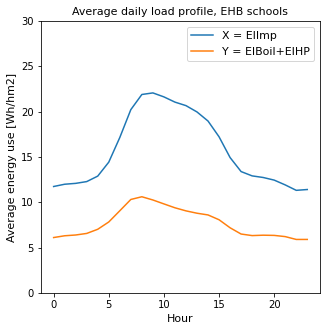  Figure 6 Average daily load profile for EHBs |
| --- | --- |

Figure 7-Figure 9 compare the average daily load profiles of EHBs and DHBs for X, Y, and the electric-specific load (X-Y), respectively in terms of total hourly energy use. The load profiles of both building types follow a similar pattern, but X and Y are slightly higher for DHBs, as previously noted. Figure 9 shows that the X-Y load curve is nearly identical for both building types, further supporting the theory that their daily load patterns are similar, but that the heating system efficiency of EHBs is higher and that they possibly have a more energy efficient building envelope.

| 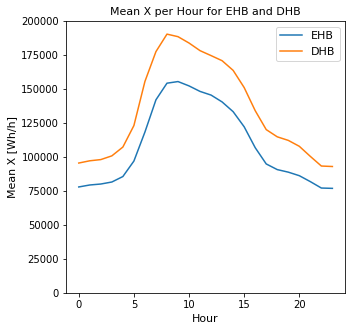  Figure 7 Comparison average daily load profile for total imported energy (X) for EHBs vs DHBs. | 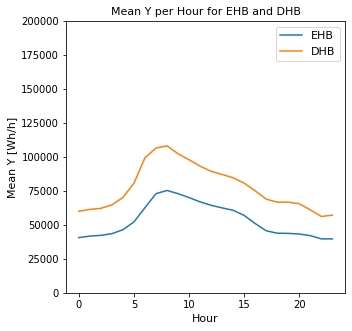  Figure 8 Comparison average daily load profile for total heating energy for EHBs vs DHBs. | 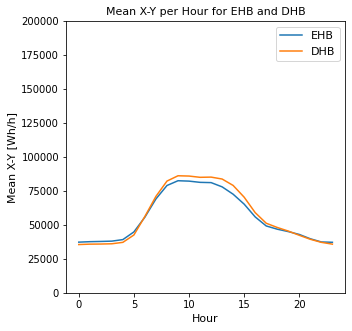  Figure 9 Comparison average daily load profile for energy for other purposes than heating (X-Y) for EHBs vs DHBs. |
| --- | --- | --- |

An Energy-Temperature (ET) plot illustrates the daily energy consumption in a building against the average daily outdoor temperature. The primary purpose of this is to show the temperature dependence of the energy consumption in the building, as well as variance in this energy consumption, which may be caused by the type of day (weekday vs. weekend) and other factors. Figure 10 and Figure 11 show the ET curves for all buildings in the DHB and EHB datasets. The figures indicate considerable variation in energy use, particularly among DHBs, which can be attributed to differences in building size, energy demand efficiency, and heating system performance. Secondly, there is a clear temperature dependence of the energy use, especially below 10-15 C. Additionally, energy use appears to be lower on weekends (WE) compared to weekdays (WD).

| 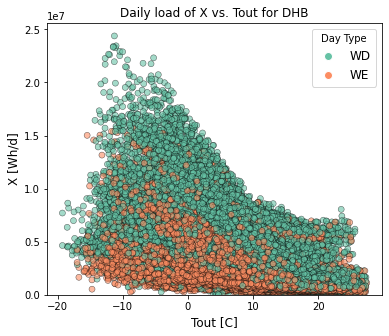  Figure 10 ET-curve for X in DHBs | 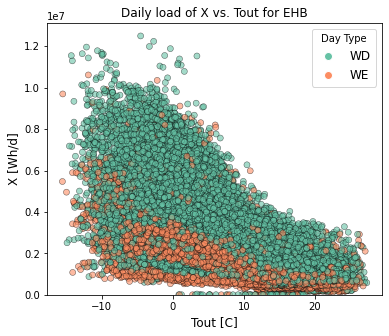  Figure 11 ET-curve for X in EHBs |
| --- | --- |

The energy signature curve (ESC) represents the relationship between the total heat load of a building and the outdoor temperature is derived from using piecewise regression on a building's ET-curve. For a typical building, the ESC consists of two distinct segments, separated by the change point temperature (CPT). The CPT is a key temperature that marks the end of the heating season when the building no longer requires energy for heating. The piecewise regression method identifies the CPT and creates separate models for the two segments of the ESC, where 𝑓(𝑥) is a model for the ESC, 𝑥 is the outdoor temperature, 𝛽0, 𝛽1, 𝛽2 are the coefficients of the piecewise model, and ε is the residual error. [43]

Equation 1

$$f\left( x \right)=\left\{ \begin{aligned} \beta_{0}+ \beta_{1}\left( x-CPT \right)+\varepsilon, &x<CPT \\ \beta_{0}+ \beta_{2}(x-CPT)+\varepsilon, &x\geq CPT \end{aligned} \right.$$

The ESC of the total energy use, X, is calculated for all the buildings individually on daily and hourly level per m2. The resulting average Energy Signature Curves for X for the DHBs and EHBs and their variables are shown in Figure 12. The ESCs shows that DHB schools on average consume more energy than EHB schools at all temperature levels, but that the energy use is pretty similar above the change point temperature which occur at around 10°C for both building types. The steeper incline in energy use for DHB schools with colder weather (B1) again supports that the DHBs have less efficient heating systems or higher heat loss compared to the EHBs.

| 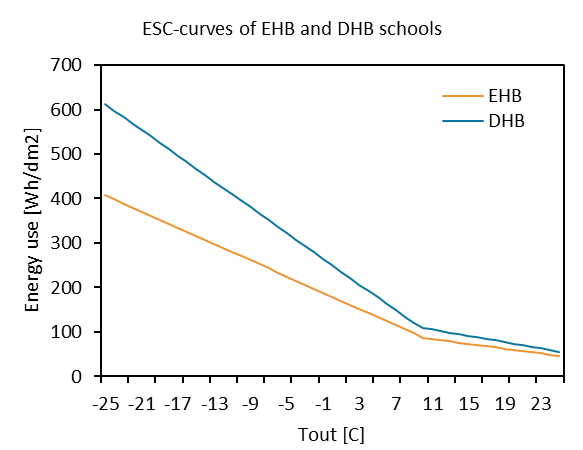 | \|  \| EHB \| DHB \| \| --- \| --- \| --- \| \| CPT \| 10.1 \| 9.6 \| \| B0 \| 86.4 \| 110.7 \| \| B1 \| -9.1 \| -14.5 \| \| B2 \| -2.7 \| -3.6 \| |
| --- | --- | --- | --- | --- | --- | --- | --- | --- | --- | --- | --- | --- | --- | --- | --- | --- |

Figure 12 ESC for X in the DHBs and EHBs.

# Results

## Step 1: Model and target evaluation

Several machine learning models are trained and tested on the different combinations of training and test sets described in Table 3 to find the best base model for the disaggregation. The only input variables used in this analysis are the original features. The target variable is either 'Y' - the energy used for heating, or 'Y/X' - the share of energy used for heating. The tested models include linear regression, random forest regression, CatBoost, and LSTM. The LSTM model relies on normalized data and is hence only evaluated for estimating ‘Y/X’ as a target. The results for all train-test-combinations, targets, and models are presented in Table 7. The model performances is evaluated on the whole test sets (not per building). This means that data from buildings in the test sets are aggregated into one continuous dataset, with the average performance measured per timestamp.

Table 7 Model results for prediction of Y and Y/X for the different models and train-test combinations.

| *Model* | *Data segment* | | *Y* | | | *Y/X* | | |
| --- | --- | --- | --- | --- | --- | --- | --- | --- |
|  | *Test* | *Train* | *R²* | *NMAE* | *PDE* | *R²* | *NMAE* | *PDE* |
| Linear regression | EHB | DHB | 0.89 | 0.032 | -33 % | 0.21 | 0.208 | -569 % |
|  |  | EHB | 0.83 | 0.042 | 32 % | 0.02 | 0.231 | -342 % |
|  |  | Both (DHB+EHB) | 0.89 | 0.032 | -14 % | 0.23 | 0.205 | -515 % |
|  | DHB | DHB | 0.85 | 0.016 | 19 % | 0.48 | 0.136 | -570 % |
| Random Forrest | EHB | **DHB** | **0.92** | **0.025** | **9 %** | 0.301 | 0.183 | 11 % |
|  |  | EHB | 0.83 | 0.039 | 7 % | 0.034 | 0.223 | 14 % |
|  |  | **Both (DHB+EHB)** | **0.91** | **0.027** | **9 %** | 0.3 | 0.184 | 11 % |
|  | DHB | DHB | 0.79 | 0.014 | -6 % | 0.443 | 0.134 | 0 % |
| CatBoost | EHB | **DHB** | **0.91** | **0.026** | **-8 %** | 0.29 | 0.185 | 13 % |
|  |  | EHB | 0.83 | 0.038 | 14 % | 0.04 | 0.221 | 10 % |
|  |  | **Both (DHB+EHB)** | **0.91** | **0.027** | **-3 %** | 0.29 | 0.185 | 14 % |
|  | DHB | DHB | 0.84 | 0.013 | 36 % | 0.45 | 0.134 | 1 % |
| LSTM | EHB | DHB | - | - | - | 0.18 | 0.203 | -34 % |
|  |  | EHB | - | - | - | 0.14 | 0.209 | 10 % |
|  |  | Both (DHB+EHB) | - | - | - | 0.23 | 0.197 | -14 % |
|  | DHB | DHB | - | - | - | 0.47 | 0.134 | -42 % |

The model results indicate better performance when using 'Y' as the target variable rather than 'Y/X'. Among the tested models, CatBoost and Random Forest Regression consistently achieve high performance across most train/test combinations, particularly when trained on DHB or DHB+EHB data to estimate 'Y' in previously unseen all-electric buildings (EHB test). Both models perform similarly in terms of R² and NMAE, with CatBoost slightly outperforming Random Forest Regression. For the PDE metric, CatBoost shows better performance compared to Random Forest in the DHB train–EHB test scenario. Given its high performance and significantly faster runtime, CatBoost is selected for further analysis.

A comparison between the measured and predicted values of 'Y' or 'Y/X' for the buildings in test EHB when trained on train DHB with the different models is shown in Figure 13-Figure 16. The results demonstrate a good fit for both the Random Forest Regression and CatBoost models, although there are some differences between them. CatBoost tends to overestimate peak loads, while Random Forest Regression more frequently underestimates the peaks.

| 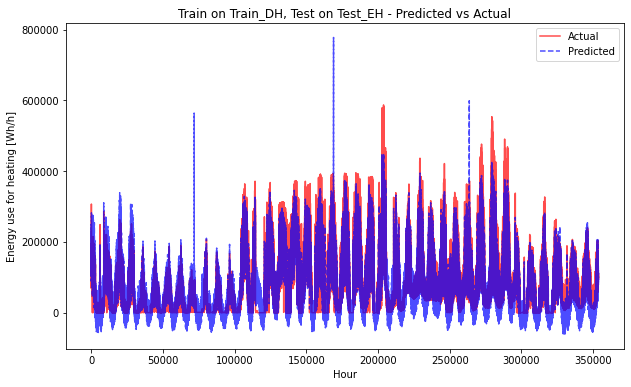  Figure 13 Linear Regression Y | 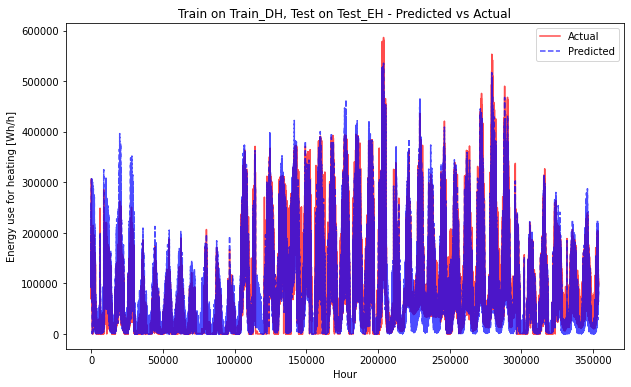  Figure 14 RandomForrestRegression Y |
| --- | --- |
| 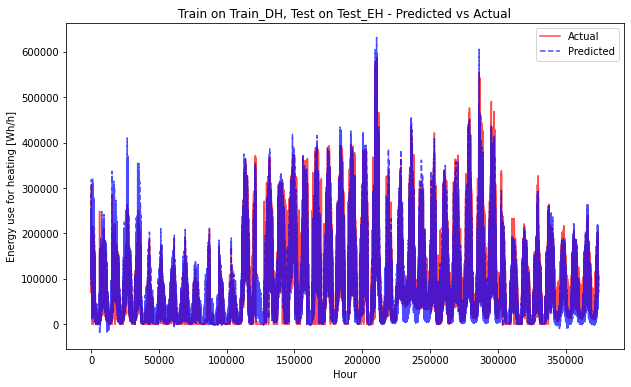  Figure 15 CatBoost Y | 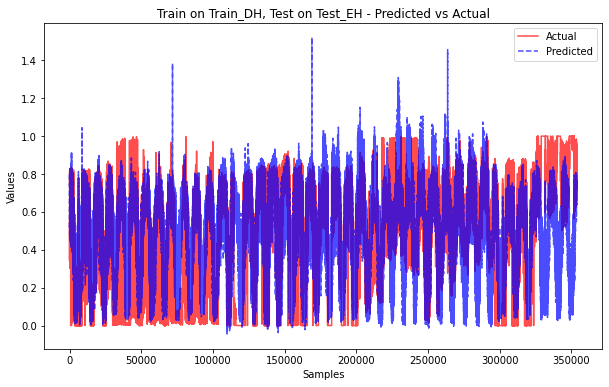  Figure 16 LSTM Y/X |

## Step 2: Building level evaluation

This section evaluates the performance of heating load disaggregation using the selected CatBoost model at the individual building level. We analyse buildings in the test sets based on various train-test-combinations. Table 8 summarizes the model's performance in predicting heating energy ("Y") for each building, with green highlights marking the best results for each individual building and red highlights indicating the worst, for the given training set.

Table 8 CatBoost model performance on the individual buildings in the test sets when the model is trained on the different training sets. Green indicates the best result for that particular building, while red indicates the worst result for the individual building based on the given performance metric.

| TEST EHB | *Train DHB* | | | | *Train EHB* | | | | *Train Both* | | | |
| --- | --- | --- | --- | --- | --- | --- | --- | --- | --- | --- | --- | --- |
|  | *Building ID* | *R²* | *NMAE* | *PDE* | *Building ID* | *R²* | *NMAE* | *PDE* | *Building ID* | *R²* | *NMAE* | *PDE* |
|  | 6416 | 0.67 | 0.056 | -33 % | 6416 | 0.73 | 0.060 | -11 % | 6416 | 0.70 | 0.056 | -30 % |
|  | 8129 | 0.39 | 0.054 | -2 % | 8129 | 0.71 | 0.035 | 19 % | 8129 | 0.60 | 0.042 | 1 % |
|  | 8098 | 0.94 | 0.044 | -10 % | 8098 | 0.82 | 0.079 | -27 % | 8098 | 0.92 | 0.049 | -12 % |
|  | 8101 | 0.91 | 0.028 | -8 % | 8101 | 0.83 | 0.043 | 14 % | 8101 | 0.91 | 0.030 | -3 % |
|  | 6408 | 0.96 | 0.025 | 10 % | 6408 | 0.81 | 0.051 | 24 % | 6408 | 0.95 | 0.030 | 9 % |
|  | 6420 | 0.68 | 0.085 | -10 % | 6420 | 0.34 | 0.132 | 9 % | 6420 | 0.62 | 0.100 | -6 % |
|  | *Average (ABS)* | *0.76* | *0.049* | *12 %* | *Average* | *0.71* | *0.067* | *17 %* | *Average* | *0.78* | *0.051* | *10 %* |
| TEST DHB | *Train DHB* | | | |  | | | | | | | |
|  | *Building ID* | *R²* | *NMAE* | *PDE* |  |  |  |  |  |  |  |  |
|  | 8014 | 0.91 | 0.034 | 13 % |  |  |  |  |  |  |  |  |
|  | 8017 | 0.90 | 0.030 | -4 % |  |  |  |  |  |  |  |  |
|  | 8035 | 0.96 | 0.026 | 3 % |  |  |  |  |  |  |  |  |
|  | 8040 | 0.94 | 0.026 | 4 % |  |  |  |  |  |  |  |  |
|  | 8054 | 0.95 | 0.022 | 7 % |  |  |  |  |  |  |  |  |
|  | 8126 | 0.95 | 0.027 | 0 % |  |  |  |  |  |  |  |  |
|  | 8005 | 0.94 | 0.021 | -8 % |  |  |  |  |  |  |  |  |
|  | 8016 | 0.90 | 0.026 | 4 % |  |  |  |  |  |  |  |  |
|  | 8114 | 0.70 | 0.036 | 36 % |  |  |  |  |  |  |  |  |
|  | *Average* | *0.91* | *0.028* | *9 %* |  |  |  |  |  |  |  |  |

The performance of predicting *Y* in the test sets varies across individual buildings, with some showing strong results while others exhibit lower accuracy in heating load disaggregation. Overall, models trained on the Train DHB or Train Both sets perform well across most metrics and buildings, with a few exceptions. In contrast, the model trained on Train EHB generally performs significantly worse, except for two cases, which will be further examined in the following subsections. On average, models trained on Train DHB or Train Both yield similar results, with an R² of 0.76–0.78 for EHB buildings and 0.91 for DHB buildings. The average NMAE is approximately 0.05, while the PDE ranges from 10% to 12% for EHB buildings when trained on DHB or Both. The average performance metrics per building differ from the overall test set averages (shown in Table 7) due to varying time series durations per building. Some buildings have a greater influence on the hourly average, contributing to this discrepancy. Figure 17-Figure 22 show the comparison between the measured and estimated "Y" values for buildings in the Test EHB set throughout the duration of the time series, while Figure 23 to Figure 28 show the predicted vs the measured value against each hour per building. The error (predicted minus measured) boxplot is given in Figure 29. Appendix B presents the measured values of *X* and *Y* for all buildings in the Test EHB set, along with details on which meters are included in *Y* and the expected accuracy of disaggregation for each building.

| 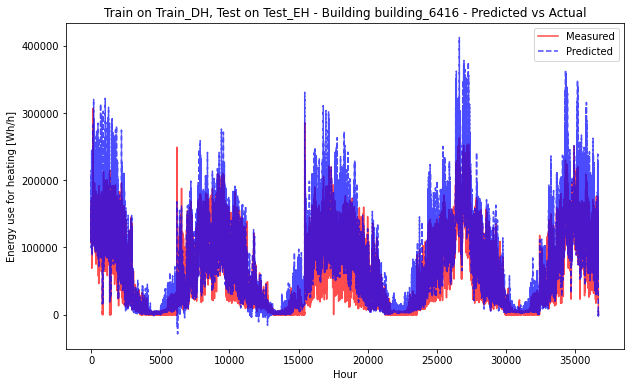  Figure 17 Measured vs. predicted Y for 6416 | 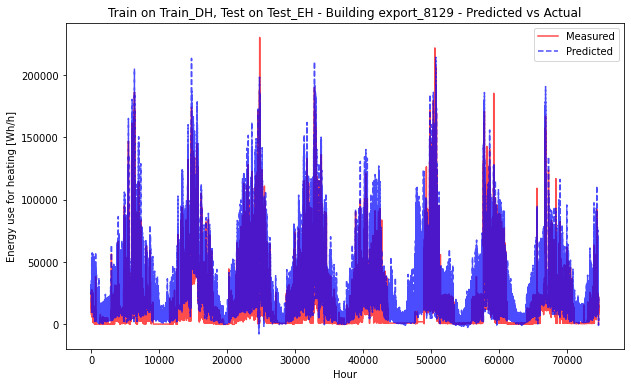  Figure 18 Measured vs. predicted Y for 8129 |
| --- | --- |
| 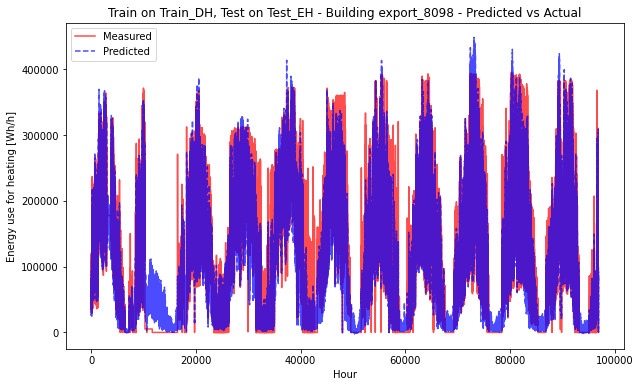  Figure 19 Measured vs. predicted Y for 8098 | 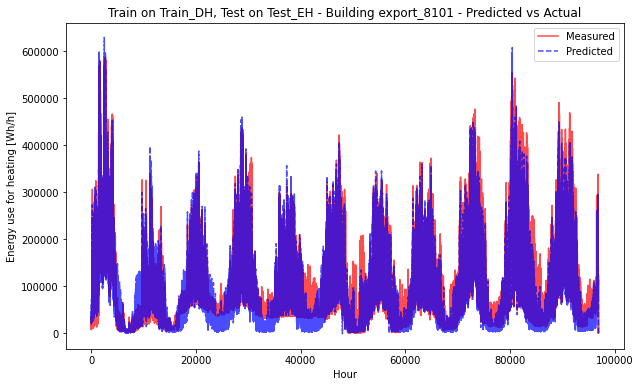  Figure 20 Measured vs. predicted Y for 8101 |
| 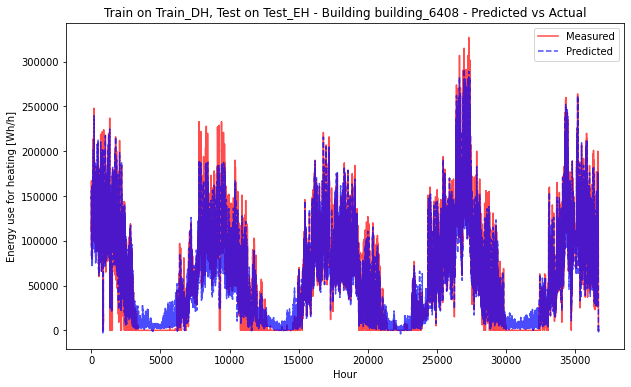  Figure 21 Measured vs. predicted Y for 6408 | 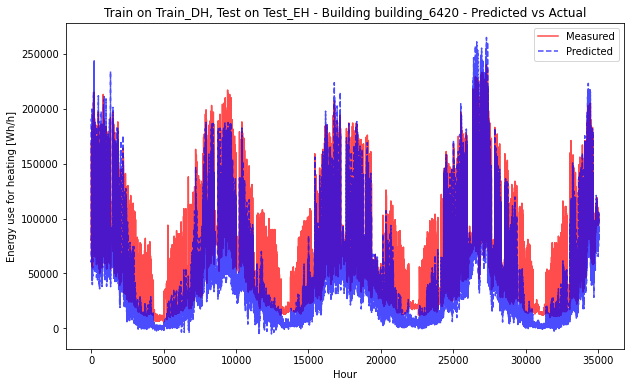  Figure 22 Measured vs. predicted Y for 6420 |

The estimated Y for Building 6416 is higher than the measured value. In this building there is an air-source heat pump for ventilation heating not included in Y as it has a missing sub-meter. It was hence to be expected that the fit for this building would be poor, and that Y would be estimated to be higher compared to the measured value. This missing sub-meter for this ventilation heating likely explains the overestimation in the disaggregated load, but unfortunately, validation is not possible.
For Building 8129, the disaggregated load shows low R² and high NMAE, primarily due to missing data for Y in summer months and longer periods, leaving several false zero values for the measured Y. However, the peak load is well estimated. The predicted value for Y is generally higher than the measured value. This discrepancy could result from either a poor fit for the disaggregation model or that there is ventilation heating in the building which is not included in the sub-meters, as in building 6416.
In Building 6420, the meter for X and Y is equal during autumn and spring which suggests that some non-heating is included in the measurements of Y. Due to this, a poor fit for the disaggregation is to be expected for 6420 during these periods. See the appendix for more details.
In contrast, Buildings 8098, 8101, and 6408 exhibit more similarities between the predicted and measured Y, with no additional unmeasured heating expected. For these buildings, there is no known issues with faulty meters or heating not accounted for (see appendix for details). A good fit for these buildings supports the hypothesis that a model trained on district heating buildings can effectively disaggregate electricity for heating in all-electric buildings.

| 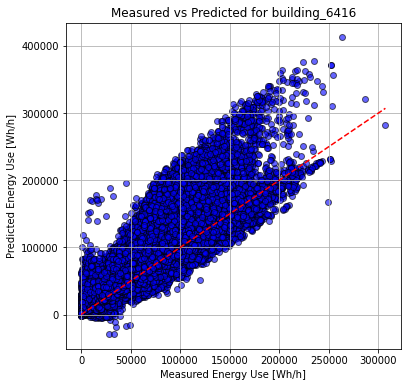  Figure 23 Measured vs. predicted energy use per hour for building ID 6416 | 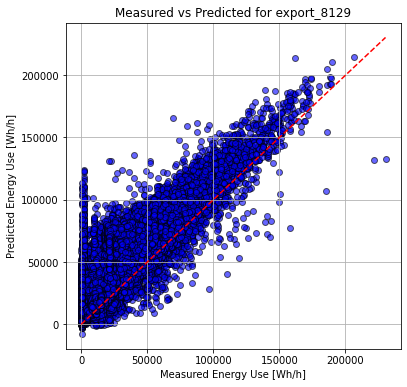  Figure 24 Measured vs. predicted energy use per hour for building ID 8129 |
| --- | --- |
| 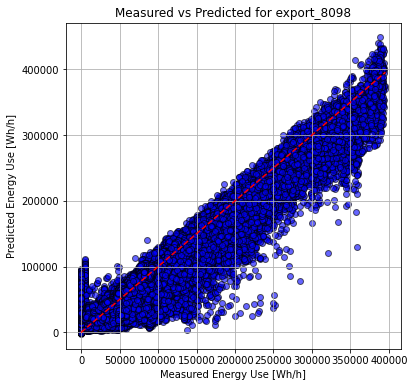  Figure 25 Measured vs. predicted energy use per hour for building ID 8098 | 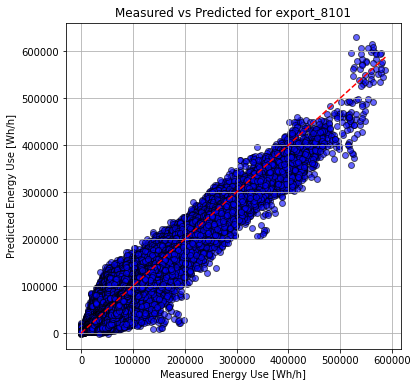  Figure 26 Measured vs. predicted energy use per hour for building ID 8101 |
| 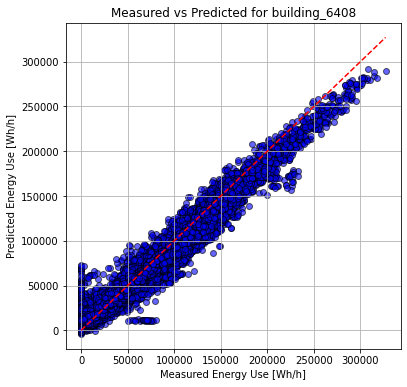  Figure 27 Measured vs. predicted energy use per hour for building ID 6408 | 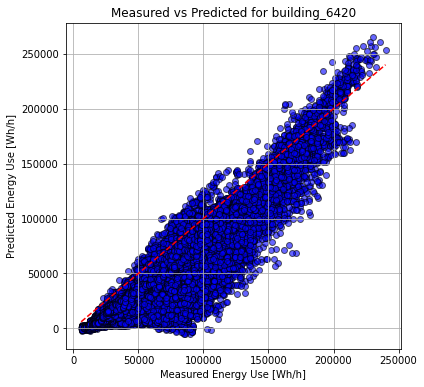  Figure 28 Measured vs. predicted energy use per hour for building ID 6420 |


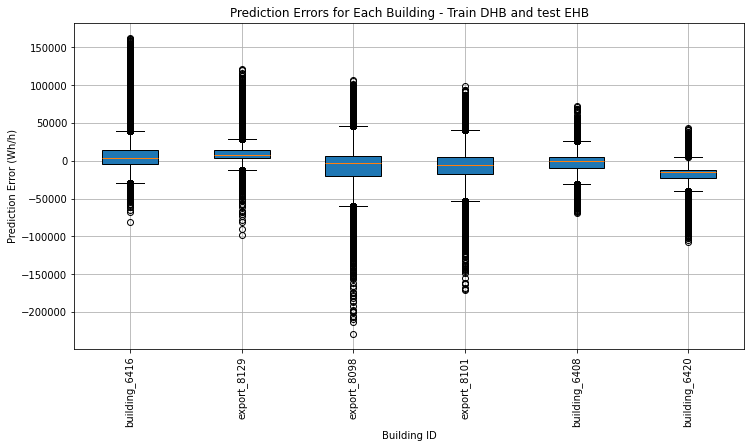


Figure 29 Error box plots – estimated load subtracted by the measured load for heating per building.

## Step 3: Feature engineering evaluation and exploration

### Feature engineering

A feature engineering approach is tested to see if this can improve the model performance, as described in 3.2. Table 9 shows the feature importance of the original features when the CatBoost model was trained on the original features and with the different training datasets. CatBoost determines feature importance using the Prediction Values Change method. This approach evaluates how much each feature contributes to improving predictions by measuring the reduction in the model's loss function when the feature is used in tree splits. The importance score is calculated by aggregating these contributions across all trees, with higher values indicating a greater impact on predictions. The scores are normalized for comparability with a sum of feature importances being 100. The table shows a very high importance for X as a feature for all train sets, which can be expected as there is a close correlation between X and Y. The table reveals a strong similarity in how features are valued when using training sets based on DHBs and Both DHBs and EHBs, which may explain the similar performance of models trained on these datasets. However, when trained on EHBs only, the model assigns less importance to outdoor temperature and greater importance to solar radiation (SolGlob). While solar radiation may serve as an indicator of school "working hours," relying too heavily on it could lead to misleading results. This is because heating demand naturally decreases in summer when solar radiation remains high throughout the day, potentially skewing the model’s predictions.

Table 9 Feature importance with original features.

| *Feature* | *Train_DHB* | *Train_EHB* | *Train_Both* | *Average Importance* |
| --- | --- | --- | --- | --- |
| Tout | 25.3 | 14.5 | 21.1 | 20.3 |
| SolGlob | 2.7 | 7.0 | 3.3 | 4.3 |
| WindSpd | 0.8 | 2.0 | 0.8 | 1.2 |
| WindDir | 2.2 | 4.1 | 1.9 | 2.8 |
| X | 68.9 | 72.4 | 72.9 | 71.4 |

Several feature combinations are included to assess how performance varies with different feature engineering approaches. These combinations consist of the 1) original features 2) The original features plus 223 features derived from the timestamp and original features, and 3) The original features plus 223 features plus additional features such as building metadata and energy transition (ET) curves for each individual building. A summary of the different feature sets is presented in Table 10

Table 10 Feature sets

| *Feature set*  *Short name* | *Number of features* | *Features* | *Description* |
| --- | --- | --- | --- |
| Orig. | 5 | Tout, SolGlob, WindSpd, WindDir, X | Original features |
| Orig + FE | 5 + 223 | Features described in Table 5 | Original features + features extracted from original features and timestamp. |
| Orig + FE  +ET-curve  +meta | 5+ 223 + 4 + 2 | FE + CPT + B0 + B1 + B2 + floor area + year of construction | Original features + 223 features extracted from original features and timestamp  + ET-curve features and meta data features are included in the feature extracted dataset. |

Table 11 presents the model's performance using the different train-test combinations and feature sets with the CatBoost model. The performance is evaluated on the full dataset evaluation level. The results indicate that, for most train-test-combinations, adding additional features only leads to a slight improvement in R² and NMAE for most buildings, while the peak demand error is worsened for most train-test combinations with increased features. For the EHB train – EHB test combination, the performance goes down in the third feature set. This may be due to overfitting to the year of construction of floor area which can be uncertain in school buildings which are built, changed and extended over the long lifespan of school buildings.

Table 11 Performance of disaggregation with feature engineering vs. original features

| *Feature set* | | *R²* | | | *NMAE* | | | *PDE* | | |
| --- | --- | --- | --- | --- | --- | --- | --- | --- | --- | --- |
|  |  | *Orig.* | *FE* | *FE*  *+ETcurve*  *+meta* | *Orig.* | *FE* | *FE +ETcurve +meta* | *Orig.* | *FE* | *FE +ETcurve +meta* |
| *Test set* | *Train Set* |  |  |  |  |  |  |  |  |  |
| EHB | DHB | 0.91 | 0.92 | 0.94 | 0.026 | 0.026 | 0.023 | 8 % | 14 % | 11 % |
|  | EHB | 0.83 | 0.83 | 0.77 | 0.038 | 0.037 | 0.042 | 14 % | 17 % | 25 % |
|  | BOTH | 0.91 | 0.91 | 0.93 | 0.027 | 0.028 | 0.024 | 3 % | 11 % | 12 % |
| DHB | DHB | 0.84 | 0.92 | 0.94 | 0.013 | 0.01 | 0.009 | 36 % | 33 % | 28 % |

Table 12 shows the building level results for the heating disaggregation in the Test EHB set when trained on the DBH-train set. The building level results do, however, show an improvement in average building performance compared to the full dataset evaluation results. Adding ET-curve features (CPT, B0, B1, and B2), year of construction and floor area can significantly improve the performance variables of some of the buildings. These are static features that are the same for all hours of the time series per building. For other buildings, however, these features can reduce performance, especially for peak demand errors.

Table 12 Per building evaluation in Test EH trained on Train DH. Comparison when training on original vs. feature-engineered- dataset.

| *Building ID* | *R²* | | | *NMAE* | | | *PDE* | | |
| --- | --- | --- | --- | --- | --- | --- | --- | --- | --- |
|  | *Orig.* | *FE* | *FE*  *+ETcurve*  *+meta* | *Orig.* | *FE* | *FE +ETcurve +meta* | *Orig._* | *FE* | *FE +ETcurve +meta* |
| 6416 | 0.67 | 0.69 | 0.81 | 0.056 | 0.056 | 0.045 | -33 % | -25 % | -17 % |
| 8129 | 0.39 | 0.46 | 0.67 | 0.054 | 0.056 | 0.035 | -2 % | 3 % | 16 % |
| 8098 | 0.94 | 0.95 | 0.96 | 0.044 | 0.039 | 0.037 | -10 % | -5 % | -7 % |
| 8101 | 0.91 | 0.89 | 0.90 | 0.028 | 0.033 | 0.031 | -8 % | 14 % | 11 % |
| 6408 | 0.96 | 0.98 | 0.98 | 0.025 | 0.019 | 0.018 | 10 % | 5 % | -1 % |
| 6420 | 0.68 | 0.7 | 0.76 | 0.085 | 0.082 | 0.075 | -10 % | -9 % | -16 % |
| *Average (ABS)* | *0.76* | *0.78* | 0.85 | *0.049* | *0.047* | 0.040 | *12 %* | *10 %* | 11 % |

Figure 30 the feature importances for the top ten most important features in the CatBoost model trained on Train DHB with the complete feature set (Orig+FE+ETCurve+Meta). The results show a reduced importance for feature X and an increased importance for outdoor temperature compared to when the model is trained on the original features. Additionally, the meta and ET-curve features are assigned relatively high importance compared to the other features in the dataset. Floor area may not have made the top 10 important features as there is some uncertainty in the validity of this number, or that energy efficiency represented by the year of construction is more important to the total energy use of the school buildings in the training set.


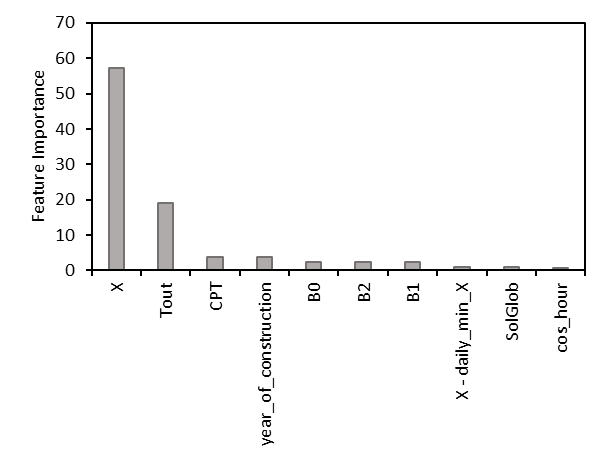


Figure 30 Feature importances for the CatBoost model trained on Train DHB with complete feature set (Orig+FE+ETCurve+Meta)

### Feature and Cross-Domain Transferability Analysis using t-SNE

To better understand the structural differences captured by the engineered features, X + FE features for each building were transformed into a single building-level feature set. For each of the 74 buildings, the 224 features were summarized using five statistical measures (mean, median, minimum, maximum, and standard deviation), resulting in a data matrix with 74 rows and 1,115 columns. This final matrix was then used to generate a two-dimensional t-SNE (t-distributed Stochastic Neighbour Embedding) projection.

t-SNE is a non-linear dimensionality reduction technique that maps high-dimensional data into a lower-dimensional space while preserving local similarities between data points[44]. It is particularly useful for visualizing complex structures in data, such as clusters or patterns that may not be apparent in the original feature space. While the resulting components do not have a direct physical meaning, the relative positioning of points reflects their similarity based on the original engineered features. However, as a lossy transformation, some information may be lost when projecting data from the high-dimensional feature space to the two-dimensional visualization. The t-SNE visualization in Figure 31 shows how buildings cluster according to heating system type (EHB vs. DHB) and geographic location, providing insight into the structural differences captured by the engineered features.


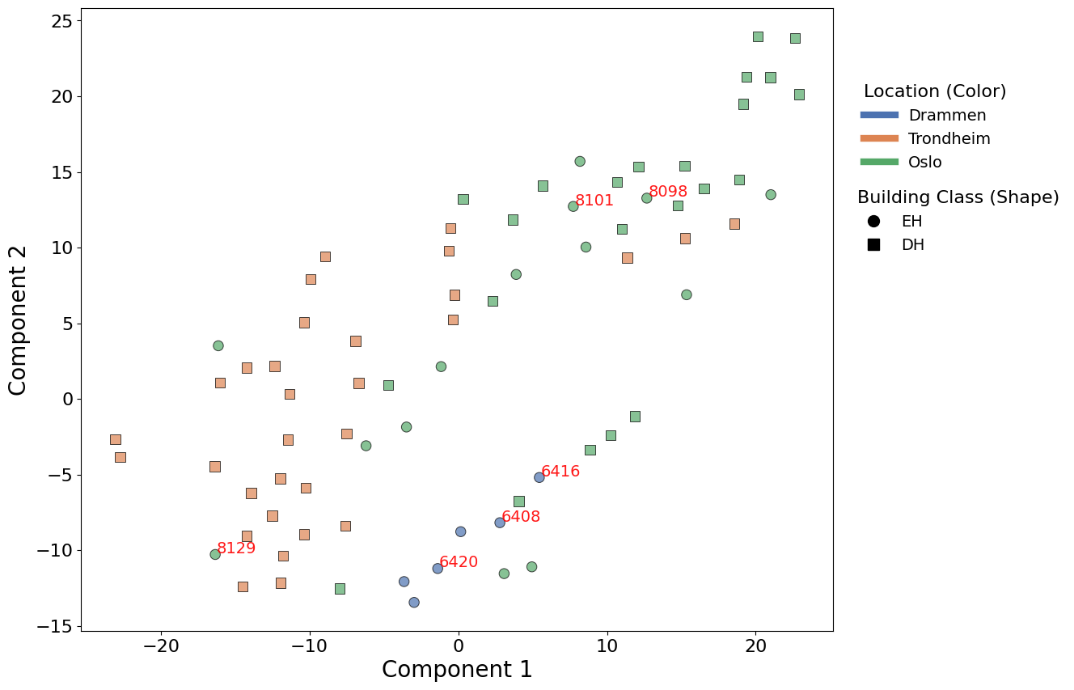


Figure 31 t-SNE 2D projections of building-level FE features, colored by location, with markers indicating building class, and red text labeling the unique ID of EHBs in the test set.

The t-SNE plot shows that EHB and DHBs are partially separable in feature space, suggesting that the engineered features capture meaningful differences in energy consumption patterns. However, some degree of overlap is also evident, likely due to shared non-heating end uses such as lighting, appliances, and ventilation. Notably, buildings tended to cluster by geographic location regardless of heating type, indicating that the feature extraction process captures regional energy use characteristics. However, variations in data duration could also play a role in shaping these clusters. For instance, Drammen data covers four years, Trondheim three years, and Oslo between one and eleven years. The length of the available time series may affect how representative the aggregated features are, particularly for buildings with shorter observation windows. Nevertheless, the strong geographic clustering indicates that regional usage patterns remain dominant, despite potential differences introduced by varying data durations.

These spatial groupings are consistent with observed model generalization patterns. EHBs that are closely embedded within DHB clusters in t-SNE space, such as buildings 8098 and 8101, exhibit strong performance when trained exclusively on DHB data (R² > 0.9, NMAE < 0.05). In contrast, buildings like 8129 (Oslo) and 6416 (Drammen), though visually proximate to DHBs, are surrounded by DHBs from different locations. These buildings exhibit lower performance when trained solely on DH data (R² = 0.39 and 0.67, respectively) but show substantial gains with EH-specific or combined training. This suggests that feature similarity alone is not always sufficient, and that alignment in both domain and geographic context may be necessary for effective model transfer. Interestingly, buildings 6408 and 6420, both EHBs from Drammen, perform best when trained on DHB data, despite being distant from DHBs in both geographic and t-SNE space, despite the training dataset containing no DHBs from Drammen, and despite the model having a good fit for 6408 but worse fit for 6420. This indicates that certain heating-related patterns can generalize across both domains and locations, even in the absence of close spatial proximity. It also suggests that the explicit heating signal present in DHB data enables the model to learn latent heating behaviours that are otherwise difficult to infer from EHB data alone.

# Discussion

This article investigates the potential for disaggregating electricity used for heating appliances in all-electric buildings, explicitly focusing on school buildings, using their hourly AMS measurements. A key objective was to assess whether a model trained on energy use data from buildings with district heating could be used for disaggregating heating energy in all-electric buildings. This approach is particularly appealing given the scarcity of sub-metered data for all-electric buildings.

*Performance of cross-domain disaggregation*
The results indicate that a model trained to disaggregate hourly heating energy consumption from the total hourly energy use (which includes both imported electricity and district heating) can, with high performance, generalize to previously unseen building data and buildings. In addition, the findings suggest that a model trained to disaggregate district heating from the combined energy use (electricity and district heating) can effectively disaggregate the electricity used for heating in all-electric school buildings from their AMS-meter data. CatBoostRegressor emerged as the best-performing model for this approach, with similar performance whether trained on district heating buildings alone (DHB-train) or a combination of district heating and electric heating buildings (EHB-train + DHB-train) at the full dataset evaluation level. However, at the individual building level, results were more variable, likely due to missing data or measurement errors in the dataset.

The performance metrics used for evaluation of the disaggregation performance R² and NMAE and the peak load estimation error, as well as a visual inspection of the graphs. While R² is very common and NMAE is somewhat common for evaluation of energy and power estimation, it is also a common practice to use quantitative metrics of performance alongside qualitative assessments[45]. Using the relative error of the single peak value, as done with the PDE, is less common in the literature.

*Test on unseen buildings for generalizability*The data for training, testing, and validation was split by buildings rather than by time. A common approach in disaggregation and time series prediction is to include all buildings in both the train and test sets, but use the first 80 % the time series duration for training, and the remaining for testing. However, separating the buildings so that none of them appeared in both train and test was chosen for several reasons. First, a time-based split could introduce bias in the model’s performance, as disaggregation may vary significantly between seasons. For example, models might perform better in winter when more electricity is used for heating compared to summer. This is further supported by the fact that outdoor temperature is one of the most critical features. Lastly, the goal of this approach is to demonstrate that the developed method can be applied to other buildings with electric heating in Norway, where the actual electricity used for heating is unknown. By introducing previously unseen buildings into the test and validation sets, we aim to show that the method is generalizable and can be effectively applied beyond the training dataset.

*Different targets for disaggregation modelling*Two different targets for disaggregation were investigated. The first approach focused on directly disaggregating the actual electricity used for heating (Y) from the AMS data in all-electric buildings (X), while the second targeted the proportion of electricity allocated to heating (Y/X). The results demonstrated improved performance with the first approach compared to the second. A likely reason for this is that the absolute energy use varies significantly between buildings and shows a strong correlation with both total energy consumption and outdoor temperature. In contrast, when using the proportion of electricity allocated to heating as the target (a value constrained between 0 and 1), the features were insufficient to fully capture variations in seasonality or account for differences in building age and size, leading to weaker performance, sometimes giving predictions even below zero and above 1. This also supports the use of a CatBoostRegressor model, as this performs well on non-normalized data.

*Feature engineering and its effects*
A feature engineering approach was tested to assess its impact on model performance. First, 223 features were extracted from the original dataset. Then, metadata features (floor area and year of construction) and parameters from the buildings' energy signature curves were included. This approach improved performance for NMAE and R² when all features (including metadata and energy signature features) were included. However, it led to a slight reduction in PDE performance in the DHB-train and EHB-test combination at the full dataset evaluation level. At the individual building level, performance evaluation on the EHB-test set showed significant improvements for the two worst-performing buildings when more features were included.
When using only the original features, total energy use (X) had the highest importance (69), followed by outdoor temperature (Tout) with an importance of 25. This indicates a strong correlation between X and Y in Norwegian school buildings. Adding more features redistributed feature importance, reducing the weights of X and Tout while increasing the influence of energy signature features, year of construction, daily minimum X, and solar radiation. The findings suggest that including more features, especially energy signature and metadata features, can enhance disaggregation performance for underperforming buildings. However, given the uncertainty in the validation data, adding more features may also increase the risk of overfitting. Also, while esc-features can be extracted from the AMS and weather data of a buildings, the floor area and year of construction is not information which is readily available for all buildings and require more data collection. Additionally, as the CatBoostRegressor algorithm involves some degree of randomization, results may vary depending on the random seed. Consequently, some changes in performance when including more features may be attributed to randomness rather than improvements in the data.

Finally, a t-SNE visualization revealed partial separation of EHB and DHB buildings in the engineered feature space, with geography emerging as a dominant clustering factor. Consequently, model transferability depends on alignment in both heating system type and geographic context, highlighting a complex interplay of location, system type, and learned feature representations. This spatial structure provides a useful proxy for assessing cross-domain and cross-location model transferability, underscoring the non-linear dynamics inherent in disaggregating heating loads from smart meter data.

*Practical implications*

The proposed method has several practical applications. Most importantly, it demonstrates that buildings with district heating can be used to train disaggregation algorithms that separate heating from total electricity use in all-electric buildings. This enables the development of accurate models without relying on rare and costly sub-metered data from buildings with electric heating.

Trained models for disaggregating electricity for heating have multiple uses. They can provide detailed insights into when and how much electricity is used for heating in individual buildings. This information can be used for demand-side flexibility which in turn can be used to reduce peak loads, lower consumer costs, and mitigate grid congestion during high-demand periods. The information can also be used for building energy ratings.

The method can also improve and extend tools for generating building load profiles. One such tool, developed in a Norwegian context, is PROFet (energy demand load profile estimator) [39], [46], which uses measured data from Norwegian buildings. This model separate thermal and non-thermal heating demand. The proposed methodology could enhance this tool by providing more load profiles from buildings without sub-meters by providing this split between thermal and non-thermal energy demand through disaggregation. It is particularly valuable for distribution system operators and energy companies to access tools that estimate typical hourly energy demand split into electric and thermal components for different building types and climate. PROFet can also be used to provide models of different areas load profiles by taking into account the building types present in the area and the climate. It can then be used to provide “what-if” scenario analyses of technology shifts, such as switching heating from electricity to district heating, helping assess the impact on both district heating and electricity grids. These tools can deliver realistic estimates of peak loads and simultaneity effects which can be used in grid planning without relying on sensitive, user-specific sub-metering data. This tool and similar tools can support grid operators in their role as neutral energy coordinators, enabling them to provide objective guidance to policymakers, regional authorities, municipalities, and businesses regarding the capabilities and limitations of the local energy system.

*Limitations of the study and unresolved uncertainty* A significant source of uncertainty lies in the validity of the total electricity use for heating in the EHB (all-electric buildings). While the quality of AMS-meters [47] and district heating meters [48] is regulated by Norwegian law, there are no such data quality requirements for submeter data [49]. There is some uncertainty in how meters are relabelled within buildings. For instance, a building may have multiple AMS meters and report using one exclusively for an electric boiler, yet other appliances may also be connected to this meter without being reported, as suspected in building 6420 from the EHB-test set. In other cases, unmeasured heating appliances, such as hot water heaters or potentially electric panel heaters, could lead to an underestimation of electricity consumption for heating. For example, in building 8112, a heat pump is known to be used for ventilation heating, but since it lacks an electricity meter, a significant portion of heating-related electricity use may not be captured. The extent of this uncertainty remains unknown. For buildings with district heating, it is generally assumed that most of the heating demand is met by the district heating system. However, some may still have unreported electric heating, such as electric floor heating or ventilation heating in certain parts of the building. There may also be variations in the data due to location. All EHBs in the dataset are situated in the Oslo area, specifically in Oslo and Drammen, whereas most DHBs are from Trondheim, which falls within a different climate zone. Still, it appears that the performance is considerable ant that the model can be used for cross domain training, both across building, heating system and location.

*Further work*This article serves as a proof of concept, demonstrating that buildings with district heating can be used to train a model to disaggregate electricity for heating from the AMS meters of all-electric buildings. Future research could explore applying this method to other building types, both residential and non-residential, as well as to different geographic locations. Additionally, investigating the model's performance at varying time resolutions and across a broader range of environments would be valuable. Future work could also explore sequence-to-point learning models with the hourly data, which have shown strong performance in other energy disaggregation tasks for various household appliances and in various studies, as presented in [50].

# Conclusion

This case study on 74 school buildings from Norway demonstrates the feasibility of using a model trained on district-heated buildings to disaggregate electricity for heating in all-electric buildings from their AMS-meter data. Results show that CatBoost achieved a high performance in disaggregating heating electricity in all-electric buildings when trained with data from DHBs, with an R² value of 0.91, NMAE of 2.6 %, and a peak load estimation error of 8 % at the full dataset evaluation level. However, at the individual building level, results were more variable, likely due to errors and uncertainty in the dataset. Feature engineering improved the disaggregation performance for underperforming buildings, especially by including of metadata features such as floor area, year of construction, and energy signature curve constants for the building. However, these features are not always readily available, which could limit the practical application of the approach without additional data collection. In addition, the presence of uncertainty in validation data suggests that increasing the number of features may introduce a risk of overfitting. Testing two model targets including the absolute heating electricity use (Y) and the proportion of electricity used for heating (Y/X) showed that predicting Y improved the model performance for all models. This is likely because absolute energy use varies significantly between buildings and correlates strongly with total energy consumption and outdoor temperature. In contrast, Y/X, constrained between 0 and 1, failed to capture the large variations between the buildings in the dataset. These findings further support the use of CatBoostRegressor or other tree or gradient boosting algorithms which performs well on non-normalized data. While striving for perfect performance remains challenging due to uncertainties in the dataset, this study provides a promising pathway to cost-effective disaggregation of electricity for heating in all-electric buildings, by training on data from buildings with district heating measurements which are more readily available.

# Acknowledgments

This article was written as part of the research project “Coincidence Factors and Peak Loads of Buildings in the Norwegian Low-Carbon Society” (COFACTOR). The authors gratefully acknowledge the support of the Research Council of Norway (project number 326891), as well as contributions from research partners, industry partners, and data providers. Special thanks go to Drammen Municipality for sharing data from the Drammen schools.

The authors also greatly acknowledge Oslobygg KF and Smart Building Hub (Research Council of Norway project number 322573) for providing data from buildings in Oslo Municipality, and to Trondheim Municipality and Trondheim Eiendom for sharing data from the buildings in Trondheim.

# Author contributions: CRediT

**Synne Krekling Lien**: Conceptualization, Data Curation, Formal analysis, Investigation, Methodology, Software, Validation, Visualization, Writing - Original Draft, Writing - Review & Editing. **Ada Canaydin:** Writing - Original Draft ,Writing - Review & Editing, Visualisation, Methodology, Investigation. **Clayton Miller**: Writing - Review & Editing, Supervision, Visualisation. **Chun Fu:** Writing - Review & Editing. **Hussain Kazmi:** Writing - Review & Editing. **Jayaprakash Rajasekharan:** Writing - Review & Editing, Supervision

# Declaration of generative AI and AI-assisted technologies in the writing process.

During the preparation of this work the authors used ChatGPT4o in order to do spelling/grammar checks as well as receiving suggestions for rephrasing of some paragraphs/sentences to improve language and readability. After using this tool, the authors reviewed and edited the content as needed and take full responsibility for the content of the publication.

References

[1] S. Mirasgedis, L. F. Cabeza, and D. Vérez, ‘Contribution of buildings climate change mitigation options to sustainable development’, *Sustainable Cities and Society*, vol. 106, p. 105355, Jul. 2024, doi: 10.1016/j.scs.2024.105355.

[2] M. Behl, F. Smarra, and R. Mangharam, ‘DR-Advisor: A data-driven demand response recommender system’, *Applied Energy*, vol. 170, pp. 30–46, May 2016, doi: 10.1016/j.apenergy.2016.02.090.

[3] H. Kazmi, M. Keijsers, F. Mehmood, and C. Miller, ‘Energy balances, thermal performance, and heat stress: Disentangling occupant behaviour and weather influences in a Dutch net-zero energy neighborhood’, *Energy and Buildings*, vol. 263, p. 112020, May 2022, doi: 10.1016/j.enbuild.2022.112020.

[4] S. Naylor, M. Gillott, and T. Lau, ‘A review of occupant-centric building control strategies to reduce building energy use’, *Renewable and Sustainable Energy Reviews*, vol. 96, pp. 1–10, Nov. 2018, doi: 10.1016/j.rser.2018.07.019.

[5] H. Kazmi, Í. Munné-Collado, F. Mehmood, T. A. Syed, and J. Driesen, ‘Towards data-driven energy communities: A review of open-source datasets, models and tools’, *Renewable and Sustainable Energy Reviews*, vol. 148, p. 111290, Sep. 2021, doi: 10.1016/j.rser.2021.111290.

[6] S. Paustian, J. Köhlke, J. Mattes, and S. Lehnhoff, ‘Ready, Set, … Rollout? – The Role of Heterogeneous Actors and Proximities in the Delayed Smart Meter Rollout in Germany’, *Cleaner Engineering and Technology*, p. 100930, Mar. 2025, doi: 10.1016/j.clet.2025.100930.

[7] A. Zoha, A. Gluhak, M. A. Imran, and S. Rajasegarar, ‘Non-Intrusive Load Monitoring Approaches for Disaggregated Energy Sensing: A Survey’, *Sensors*, vol. 12, no. 12, Art. no. 12, Dec. 2012, doi: 10.3390/s121216838.

[8] J. Z. Kolter and T. Jaakkola, ‘Approximate Inference in Additive Factorial HMMs with Application to Energy Disaggregation’, in *Proceedings of the Fifteenth International Conference on Artificial Intelligence and Statistics*, PMLR, Mar. 2012, pp. 1472–1482. Accessed: Feb. 28, 2025. [Online]. Available: https://proceedings.mlr.press/v22/zico12.html

[9] J. Z. Kolter, S. Batra, and A. Y. Ng, ‘Energy disaggregation via discriminative sparse coding | Proceedings of the 24th International Conference on Neural Information Processing Systems - Volume 1’, in *NIPS’10: Proceedings of the 24th International Conference on Neural Information Processing Systems*, in 1153-1161, vol. 1. doi: 10.5555/2997189.2997318.

[10] A. Morch, H. Sæle, N. Feilberg, and K. B. Lindberg, ‘Method for development and segmentation of load profiles for different final customers and appliances’, *ECEEE 2013 Summer Studies Proceedings*, 2013, Accessed: Mar. 20, 2023. [Online]. Available: https://www.eceee.org/library/conference_proceedings/eceee_Summer_Studies/2013/7-monitoring-and-evaluation/method-for-development-and-segmentation-of-load-profiles-for-different-final-customers-and-appliances/

[11] G. Ciulla and A. D’Amico, ‘Building energy performance forecasting: A multiple linear regression approach’, *Applied Energy*, vol. 253, p. 113500, Nov. 2019, doi: 10.1016/j.apenergy.2019.113500.

[12] M. Mottahedi, A. Mohammadpour, S. S. Amiri, D. Riley, and S. Asadi, ‘Multi-linear Regression Models to Predict the Annual Energy Consumption of an Office Building with Different Shapes’, *Procedia Engineering*, vol. 118, pp. 622–629, Jan. 2015, doi: 10.1016/j.proeng.2015.08.495.

[13] M. Amayri, C. S. Silva, H. Pombeiro, and S. Ploix, ‘Flexibility characterization of residential electricity consumption: A machine learning approach’, *Sustainable Energy, Grids and Networks*, vol. 32, p. 100801, 2022.

[14] Z. Xiao, W. Gang, J. Yuan, Y. Zhang, and C. Fan, ‘Cooling load disaggregation using a NILM method based on random forest for smart buildings’, *Sustainable Cities and Society*, vol. 74, p. 103202, 2021.

[15] S. Wang, L. Du, and Q. Zhou, ‘A Semi-Supervised Deep Transfer Learning Architecture for Energy Disaggregation’, in *2019 IEEE Power & Energy Society General Meeting (PESGM)*, Aug. 2019, pp. 1–5. doi: 10.1109/PESGM40551.2019.8973556.

[16] M. Kaselimi, N. Doulamis, A. Doulamis, A. Voulodimos, and E. Protopapadakis, ‘Bayesian-optimized Bidirectional LSTM Regression Model for Non-intrusive Load Monitoring’, in *ICASSP 2019 - 2019 IEEE International Conference on Acoustics, Speech and Signal Processing (ICASSP)*, Brighton, United Kingdom: IEEE, May 2019, pp. 2747–2751. doi: 10.1109/ICASSP.2019.8683110.

[17] M. Xia, W. Liu, K. Wang, W. Song, C. Chen, and Y. Li, ‘Non-intrusive load disaggregation based on composite deep long short-term memory network’, *Expert Systems with Applications*, vol. 160, p. 113669, Dec. 2020, doi: 10.1016/j.eswa.2020.113669.

[18] S. S. Hosseini, B. Delcroix, N. Henao, K. Agbossou, and S. Kelouwani, ‘A case study on obstacles to feasible NILM solutions for energy disaggregation in quebec residences’, in *Proceedings of the 9th ACM International Conference on Systems for Energy-Efficient Buildings, Cities, and Transportation*, Boston Massachusetts: ACM, Nov. 2022, pp. 363–367. doi: 10.1145/3563357.3566151.

[19] N. Kianpoor, B. Hoff, and T. Østrem, ‘Deep Adaptive Ensemble Filter for Non-Intrusive Residential Load Monitoring’, *Sensors*, vol. 23, no. 4, Art. no. 4, Jan. 2023, doi: 10.3390/s23041992.

[20] S. K. Lien, B. Najafi, and J. Rajasekharan, ‘Advances in Machine-Learning Based Disaggregation of Building Heating Loads: A Review’, *Advances in Machine-Learning Based Disaggregation of Building Heating Loads: A Review*, 2023, Accessed: May 15, 2024. [Online]. Available: https://ntnuopen.ntnu.no/ntnu-xmlui/handle/11250/3109250

[21] J. Depoortere, J. Driesen, J. Suykens, and H. S. Kazmi, ‘SolNet: Open-source deep learning models for photovoltaic power forecasting across the globe’, May 30, 2024, *arXiv*: arXiv:2405.14472. doi: 10.48550/arXiv.2405.14472.

[22] H. Kazmi, J. Suykens, and J. Driesen, ‘Large-Scale Transfer Learning For Data-Driven Modelling Of Hot Water Systems’, presented at the Building Simulation 2019, Rome, Italy, pp. 2611–2618. doi: 10.26868/25222708.2019.210352.

[23] R. Spencer, S. Ranathunga, M. Boulic, Andries, van Heerden, and T. Susnjak, ‘Transfer Learning on Transformers for Building Energy Consumption Forecasting -- A Comparative Study’, Oct. 18, 2024, *arXiv*: arXiv:2410.14107. Accessed: Oct. 28, 2024. [Online]. Available: http://arxiv.org/abs/2410.14107

[24] M. D’Incecco, S. Squartini, and M. Zhong, ‘Transfer Learning for Non-Intrusive Load Monitoring’, *IEEE Trans. Smart Grid*, vol. 11, no. 2, pp. 1419–1429, Mar. 2020, doi: 10.1109/TSG.2019.2938068.

[25] D. Murray, L. Stankovic, and V. Stankovic, ‘REFIT: Electrical Load Measurements’. University of Strathclyde. doi: 10.15129/31da3ece-f902-4e95-a093-e0a9536983c4.

[26] J. Z. Kolter and M. J. Johnson, ‘REDD: A Public Data Set for Energy Disaggregation’, presented at the Workshop on data mining applications in sustainability (SIGKDD), San Diego, CA, pp. 59–62.

[27] J. Kelly and W. Knottenbelt, ‘The UK-DALE dataset, domestic appliance-level electricity demand and whole-house demand from five UK homes’, *Sci Data*, vol. 2, no. 1, p. 150007, Mar. 2015, doi: 10.1038/sdata.2015.7.

[28] S. Chouchene, M. Amayri, and N. Bouguila, ‘Sparse coding-based transfer learning for energy disaggregation’, *Energy and Buildings*, vol. 320, p. 114498, Oct. 2024, doi: 10.1016/j.enbuild.2024.114498.

[29] M. Khodayar, J. Wang, and Z. Wang, ‘Energy Disaggregation via Deep Temporal Dictionary Learning’, *IEEE Transactions on Neural Networks and Learning Systems*, vol. 31, no. 5, pp. 1696–1709, May 2020, doi: 10.1109/TNNLS.2019.2921952.

[30] SSB 13276, ‘Table 13276: Energibalansen. Vedforbruk i fritidsboliger, etter fyringsteknologi og landsdel 2020 - 2021’. Accessed: Oct. 13, 2022. [Online]. Available: https://www.ssb.no/statbank/table/13276/

[31] ‘Varmepumper reduserer utgiftene til strømavhengige nordmenn’, SSB. Accessed: Oct. 24, 2024. [Online]. Available: https://www.ssb.no/energi-og-industri/energi/artikler/varmepumper-reduserer-utgiftene-til-stromavhengige-nordmenn

[32] K. B. Lindberg, ‘Impact of Zero Energy Buildings on the Power System’, p. 192.

[33] Buvik, Magnus, Cabrol, Julien, Spilde, Dag, Skansaar, Ellen, Roos, Alexandra, and Grytli, Åsa, ‘NVE Rapport nr. 20/2022 Norsk og nordisk effektbalanse fram mot 2030’, Accessed: Oct. 25, 2024. [Online]. Available: https://publikasjoner.nve.no/rapport/2022/rapport2022_20.pdf

[34] L. Ødegården and S. Bhantana, *Status og prognoser for kraftsystemet 2018 rapportnr. 103-2018*. NVE, 2018. Accessed: May 31, 2023. [Online]. Available: http://publikasjoner.nve.no/rapport/2018/rapport2018_103.pdf

[35] F. Plaum, R. Ahmadiahangar, A. Rosin, and J. Kilter, ‘Aggregated demand-side energy flexibility: A comprehensive review on characterization, forecasting and market prospects’, *Energy Reports*, vol. 8, pp. 9344–9362, Nov. 2022, doi: 10.1016/j.egyr.2022.07.038.

[36] A. J. Marszal-Pomianowska, H. Johra, T. Weiss, and A. Knotzer, ‘EBC Annex 67. Characterization of energy flexibility in buildings’, Danish Technological Institute, Taastrup, 2019.

[37] S. K. Lien, B. Ludvigsen, H. T. Walnum, A. Yang, Å. L. Sørensen, and K. H. Andersen, ‘COFACTOR-SBHUB Oslo: Hourly Sub-Metered Energy Use Data from 48 public School Buildings in Oslo, Norway’, *[Submitted to] Data in Brief*, Apr. 2025.

[38] S. K. Lien, H. T. Walnum, and Å. L. Sørensen, ‘COFACTOR Drammen dataset - 4 years of hourly energy use data from 45 public buildings in Drammen, Norway’, *Sci Data*, vol. 12, no. 1, p. 393, Mar. 2025, doi: 10.1038/s41597-025-04708-3.

[39] A. Mohammadabadi, I. Sartori, and L. Georges, ‘Validation of the Energy Demand Load Profile Estimator “PROFet” for Trondheim Non-residential Buildings’, *E3S Web of Conf.*, vol. 562, p. 11002, 2024, doi: 10.1051/e3sconf/202456211002.

[40] S. K. Lien, B. Ludvigsen, H. T. Walnum, K. H. Andersen, A. Yang, and Å. L. Sørensen, ‘Hourly Sub-Metered Energy Use Data from 48 public School Buildings in Oslo’, *[Submitted to] Data in Brief*, 2025.

[41] S. K. Lien, H. T. Walnum, and Å. L. Sørensen, ‘COFACTOR Drammen dataset. 4 years of hourly energy use data from 45 public buildings in Drammen, Norway’, *Nature Scientific Data*, May 2024.

[42] J. Lin, E. Keogh, S. Lonardi, and B. Chiu, ‘A symbolic representation of time series, with implications for streaming algorithms’, in *Proceedings of the 8th ACM SIGMOD workshop on Research issues in data mining and knowledge discovery*, in DMKD ’03. New York, NY, USA: Association for Computing Machinery, Jun. 2003, pp. 2–11. doi: 10.1145/882082.882086.

[43] S. K. Lien, D. Ivanko, and I. Sartori, *Domestic hot water decomposition from measured total heat load in Norwegian buildings*. SINTEF Academic Press, 2020. Accessed: Jan. 17, 2023. [Online]. Available: https://ntnuopen.ntnu.no/ntnu-xmlui/handle/11250/2684373

[44] L. van der Maaten and G. Hinton, ‘Visualizing Data using t-SNE’, *Journal of Machine Learning Research*, vol. 9, no. 86, pp. 2579–2605, 2008.

[45] H. Johra, M. Schaffer, G. Chaudhary, H. S. Kazmi, J. Le Dréau, and S. Petersen, ‘What metrics does the building energy performance community use to compare dynamic models?’, presented at the 2023 Building Simulation Conference, Sep. 2023. doi: 10.26868/25222708.2023.1309.

[46] K. Heimar Andersen, S. Krekling Lien, K. Byskov Lindberg, H. Taxt Walnum, and I. Sartori, ‘Further development and validation of the “PROFet” energy demand load profiles estimator’, presented at the 2021 Building Simulation Conference, Sep. 2021. doi: 10.26868/25222708.2021.30159.

[47] Lovdata, *Forskrift om krav til elektrisitetsmålere*, vol. FOR-2007-12-28-1753. 2008. Accessed: Mar. 15, 2024. [Online]. Available: https://lovdata.no/dokument/SF/forskrift/2007-12-28-1753

[48] *Forskrift om krav til varmeenergimålere*, vol. FOR-2007-12-21-1743. 2008. Accessed: Mar. 15, 2024. [Online]. Available: https://lovdata.no/dokument/SF/forskrift/2007-12-21-1743

[49] DNV and RME, ‘RME Ekstern rapport Nr. 7/2022 Split responsibility and submetering requirements. Utredning og evaluering av modell for aggregering og bruk av submålere.’, May 2022.

[50] T. T. Kuzengurira, W. Guangfen, and L. Yilin, ‘Sequence-to-point learning methods for Non Intrusive Load Disaggregation: A Review’, in *2024 6th International Conference on Energy, Power and Grid (ICEPG)*, Sep. 2024, pp. 202–206. doi: 10.1109/ICEPG63230.2024.10775789.

**Appendix A: EHB Test Buildings overview**

This appendix provides an overview of the buildings in the EHB test set, including details about their heating systems, plotted values for X and Y, and expectations for disaggregation performance.

Each building's installed heating systems are specified using the following abbreviations:

- EB = Electric boiler
- HWH = Hot water heater
- GSHP = Ground source heat pump
- EHB = Electric heating battery for ventilation heating
- Ukn = Unknown

These systems serve different heating purposes:

- DHW = Domestic hot water heating
- SH = Space heating
- VENT = Ventilation heating

The target columns indicate which meters are included in Y (the target). Additionally, an assessment is provided regarding the expected disaggregation performance based on the quality and composition of the meters in Y.

More information about the data and datasets are given in [38] for the Drammen buildings and in [40] for the Oslo buildings.

| 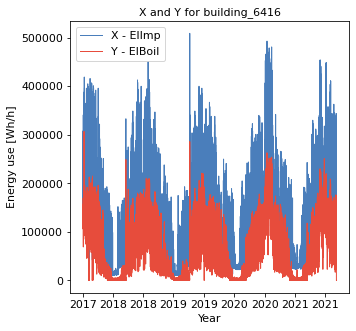 **ID:** 6416 **Location:** Drammen Y**ear of construction** 2001 **Floor area**: 8513 **Heating DHW:** EB, HWH **Heating SH**: EB **Heating VENT**: ASHP **Target meters:** ElBoil **Expectations:** Estimated disaggregation higher than measured due to missing loads for ventilation heating. | 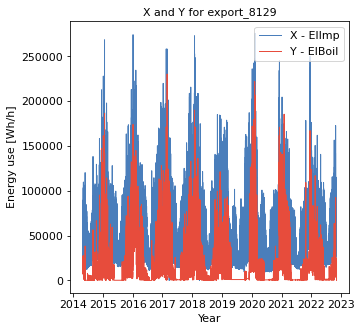  **ID:** 8129  **Location:** Oslo Y**ear of construction**: 1981 **Floor area:** 4723 **Heating DHW:** GSHP, EB  **Heating SH:** GSHP, EB  **Heating VENT:** Ukn  **Target meters**: ElHP, ElBoil Expectations: Good fit for the disaggregation. |
| --- | --- |
| 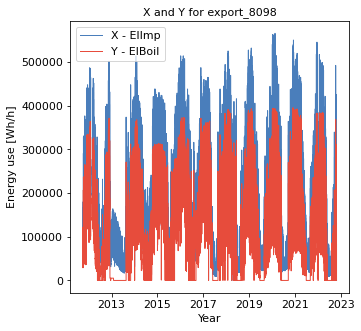 **ID:** 8098 **Location:** Oslo Y**ear of construction** : 1900 **Floor area**: 6736 **Heating DHW:** EB **Heating SH:** EB **Heating VENT:** Ukn  **Target meters:** ElBoil **Expectations**: Good fit for the disaggregation, but missing data for Y in 2013. | 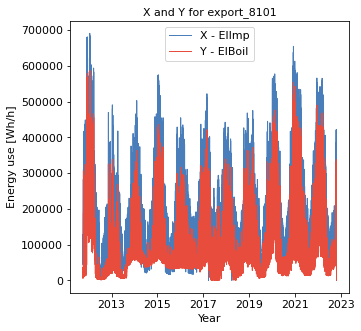 **ID:** 8101 **Location:** Oslo Y**ear of construction**: 1954 **Floor area:** 11 810 **Heating DHW:** GSHP, EB **Heating SH:** GSHP, EB **Heating VENT:** Ukn **Target meters:** ElHP, ElBoil **Expectations:** Good fit for the disaggregation. |
| 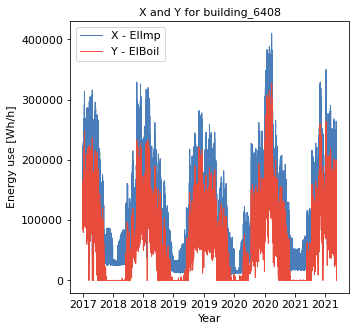 **ID:** 6408 **Location:** Drammen Y**ear of construction**: 1907 **Floor area**: 5273 **Heating DHW**: EB, HWH **Heating SH**: EB **Heating VENT**: EB **Target meters:** ElBoil **Expectations:** Good fit for the disaggregation, but slightly higher for the estimated data due to missing meter for HWH. | 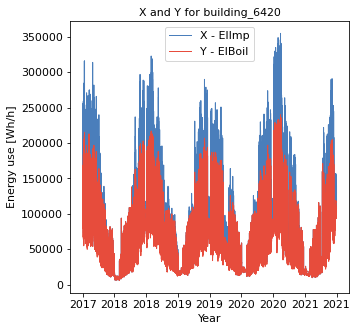  **ID:** 6420  **Location:** Drammen  Y**ear of construction**: 1969  **Floor area**: 6290  **Heating DHW**: EB, HWH  **Heating SH**: EH, EB  **Heating VENT**: EHB  **Target meters:** ElBoil **Expectations:** Missing meter for HWH and Electric heating battery. X and Y being equal during autumn/spring indicated that there may be some meter errors, and that parts of the non-heating electricity use is included in Y. Expected poor fit in these time periods for a disaggregation. |
